# The roles of MAPK signaling pathway in ovarian folliculogenesis

**DOI:** 10.1186/s13048-025-01737-9

**Published:** 2025-07-14

**Authors:** Hong Zhao, Trang Huyen Dinh, Yifu Wang, Yihua Yang

**Affiliations:** 1 Guangxi Reproductive Medical Center, The First Affiliated Hospital of Guangxi Medical University, Shuangyong Road 6, Nanning, 530021 China; 2 Key Laboratory of Early Prevention and Treatment for Regional High Frequency Tumor (Guangxi Medical University), Ministry of Education, Nanning, 530021 China; 3 Department of Urology, The First Affiliated Hospital of Guangxi Medical University, Shuangyong Road 6, Nanning, 530021 China

**Keywords:** Folliculogenesis, Follicular development, MAPK, Ovarian aging, Premature ovarian insufficiency (POI), Polycystic ovary syndrome (PCOS), Ovarian hyperstimulation syndrome (OHSS)

## Abstract

Ovarian folliculogenesis is a complex biological process critical for female fertility, intricately regulated by various signaling pathways, among which the Mitogen-Activated Protein Kinase (MAPK) signaling pathway plays a pivotal role. This review provides a comprehensive overview of the physiological functions of MAPK signaling in key stages of folliculogenesis, including primordial follicle formation and activation, dominant follicle selection, cumulus-oocyte complex (COC) expansion, ovulation, and luteinization. The orchestrating roles of MAPK on steroidogenesis and ovarian cell death are also delineated, highlighting its essential contributions to normal reproductive function. Furthermore, we explore the implications of dysregulated MAPK signaling in ovarian aging, primary ovarian insufficiency (POI), polycystic ovary syndrome (PCOS), and ovarian hyperstimulation syndrome (OHSS). By elucidating the multifaceted roles of MAPK signaling in ovarian biology, this review aims to enhance our understanding of folliculogenesis and its associated pathologies, paving the way for future research and therapeutic interventions targeting MAPK pathways in reproductive health.

**Clinical trial number** Not applicable.

## Introduction

Follicular development is a complex biological process that occurs sequentially under the precise regulation of hormones, ovarian regulatory factors, and intercellular interactions [1]. During early embryonic development, primordial germ cells are established and migrate to the future gonadal region, proliferating to form nests of cells that ultimately develop into primordial follicles [2]. The migration and homing of these primordial germ cells within the genital ridge require the coordinated regulation of various factors, including oocyte-derived peptide growth factors, growth differentiation factor 9 (GDF-9), and bone morphogenetic proteins [3]. Most follicles remain dormant, with only a few being activated to join the pool of growing follicles. The transition from primordial follicles to primary follicles, and subsequently to secondary follicles, culminates in pre-ovulatory follicles. This progression involves oocyte growth, proliferation of granulosa cells (GCs), and the formation of theca cells (TCs) [4]. Follicular steroidogenesis depends on pituitary gonadotropins, including follicle-stimulating hormone (FSH) and luteinizing hormone (LH). During each reproductive cycle, only a subset of follicles develops into dominant follicles, which are more sensitive to LH and ultimately undergo ovulation [5]. Ovulation represents the final stage of follicular development, involving meiosis resumption in the oocyte, expansion of cumulus cells, rupture of the follicle, and the release of the cumulus-oocyte complex(COC) containing a fertilizable oocyte [6]. Post-ovulation, the remaining GCs and TCs undergo terminal differentiation to form the corpus luteum (CL), which secretes progesterone to sustain pregnancy [7]. Most follicles will eventually undergo atresia, a natural regression phenomenon during follicular development [8]. Concurrently, various cell death pathways permeate the process from primordial germ cell migration to follicular atresia, contributing to the establishment of normal ovarian reserve, selection of dominant follicles, and degeneration of the corpus luteum [9].

In addition to the widely studied intercellular interactions among ovarian cells, various signaling pathways coordinate the entire process from primordial germ cell migration to follicular atresia, as summarized by researchers. The PI3K/AKT/FOXO3 signaling pathway regulates the activation of primordial follicles through *FOXO3* [10]. The nucleocytoplasmic shuttling of *FOXO3* plays a critical role in follicle activation, with its phosphorylation status influencing its localization and transcriptional activity within the nucleus [11]. The WNT signaling pathway regulates various cellular processes, including proliferation, differentiation, and apoptosis, via both β-catenin-dependent and independent mechanisms. *Wnt2* and *Wnt4* are particularly vital in follicular development, as they regulate GCs proliferation through β-catenin [12]. The Notch signaling pathway not only participates in ovarian angiogenesis but also interacts with *FOXO3*, potentially modulating follicular development through its influence on *FOXO3* [13]. In the ovary, Hedgehog signaling operates through communication between GCs and TCs, with excessive activation leading to ovulatory dysfunction and abnormal ovarian vascular development [14]. The evolutionarily conserved intracellular signaling cascade known as the MAPK (mitogen-activated protein kinase) pathway has increasingly come into focus, playing a significant role in both the physiological and pathological processes within the ovary. This review will delve into the intricate roles of the MAPK signaling pathway in ovarian function and dysfunction.

The MAPK signaling pathway is a highly conserved signal transduction cascade that is widely present from yeast to humans [15]. It plays a critical role in regulating various biological activities, including cell growth, differentiation, apoptosis, and reproduction [16, 17]. The upstream regulatory components of the MAPK pathway are diverse and complex, encompassing Rho family GTPases, the TNF receptor family, IL-1 receptors, and TLR family receptors. Key upstream activators include receptor tyrosine kinases (RTKs), G protein-coupled receptors (GPCRs), and ion channels, which activate Ras(rat sarcoma) kinases through adaptor proteins such as *Grb2* and *SOS*, initiating a cascade of phosphorylation events that sequentially activate MAPKKKs, MAPKKs, and ultimately MAPKs [18].

MAPKKKs are Ser/Thr protein kinases, usually activated by phosphorylation or interaction with small GTP-binding proteins from the Ras or Rho family. The activation of MAPKKKs leads to the phosphorylation and activation of MAPKKs, which, in turn, activate MAPKs through dual phosphorylation of specific threonine and tyrosine residues on the MAPK [19–21]. The MAPK family primarily consists of three subfamilies: ERK (Extracellular Signal-Regulated Kinases), JNK (c-Jun N-terminal Kinases), and p38 protein kinases [20]. The activation of MAPKs requires dual phosphorylation on a specific tripeptide motif (Thr-X-Tyr) located within the kinase activation loop (T-loop) [22]. MAPKs can regulate various processes through both transcription-dependent and transcription-independent mechanisms. The integration of signals and spatiotemporal regulation are influenced by multiple factors, including the cellular localization of MAPKs and their substrates, interactions with scaffold proteins, and the dephosphorylation activity of MAPK phosphatases [23]. Activated MAPKs further phosphorylate downstream effectors, which may include membrane proteins, cytoplasmic proteins, and nuclear proteins [24]. Notably, MAPKs can directly phosphorylate a variety of transcription factors, such as *AP-1*,* p53*,* c-Fos*, and *c-Jun*, thereby modulating their activity and stability, which in turn affects the expression of downstream genes [25].

The activities of ERK1 and ERK2 are regulated by upstream kinases and phosphatases, which are particularly important in female fertility [26]. The ERK pathway is the most extensively studied MAPK pathway, involving the Raf-MEK-ERK cascade [27]. Members of the Raf family, such as *Raf-1*,* B-Raf*, and *A-Raf*, activate MAPK/ERK kinases 1 and 2 (MEK1/2), which act as dual-specificity protein kinases (DUSPs) to phosphorylate specific sites on ERK1/2 [28]. Cyclic AMP (cAMP) can activate *B-Raf* through *Rap1* while inhibiting *Raf-1*, thereby influencing the activation of the MAPK pathway [28]. The ERK pathway promotes cell proliferation by activating transcription factors such as *Elk-1* and *c-Fos*, and it also regulates the transition from the G1 to S phase by affecting the expression of Cyclin D1 and the activity of *Cdk2* [29].

The JNK pathway is associated with various physiological processes and involves multiple kinases, such as MEKK, MLK, and ASK. These kinases phosphorylate and activate MAPKKs (such as MEK4/7), which subsequently phosphorylate and activate JNK, thus initiating or modulating intracellular signaling processes [30]. JNK binds to and phosphorylates c-Jun, enhancing its transcriptional activity [31]; *c-Jun* is a component of the *AP-1* transcription complex that regulates the expression of numerous cytokine genes [32].

The p38 pathway responds to cellular stress and is involved in the transmission of apoptosis and survival signals, featuring at least four distinct p38 MAPK isoforms (p38α, β, γ, and δ) [33–36]. The p38 MAPK pathway can be activated by various cellular stress conditions, including UV irradiation, heat shock, and certain mitogens [17, 37, 38]. MAPKKs (MEK3/6) activate p38 MAPKs, forming a cascading response. Notably, the activation of p38α involves a non-traditional mechanism, where TAB1 (TGF-β activated kinase 1 binding protein 1) acts as an adaptor or scaffold protein in the activation process, rather than through the phosphorylation by MKKs [39].

In addition to the three traditional MAPKs, several atypical MAPKs have been identified in recent years, including ERK3/4, ERK7/8, and NLK (Nemo-like kinase) [40]. The activation mechanisms of these atypical MAPKs differ from those of conventional MAPKs; for instance, ERK3/4 and NLK lack the Thr-X-Tyr motif typically required for phosphorylation [40]. Targeted knockout of the ERK3 gene has been shown to result in intrauterine growth restriction and early neonatal mortality [41]. Moreover, ERK7 and ERK8 may play critical roles in regulating cell proliferation and responses to estrogen [42] and glucocorticoids [43]. In *Caenorhabditis elegans*, NLK activates the TCF/LEF transcription factor POP1 through TAK-1, phosphorylating it and inhibiting its activity, which promotes the establishment of the anterior-posterior axis in a Wnt-dependent manner [44]. However, the roles of atypical MAPKs in follicular development remain to be characterized.

Previous reviews have highlighted the multifaceted roles of the MAPK pathway in male reproductive function [27], emphasizing that the regulation of ERK1/2 activity is crucial for the production and secretion of pituitary gonadotropins [45]. Currently, our understanding of the role of MAPKs in follicular development is still incomplete. This review aims to contribute to future research designs, enabling better management of infertility associated with abnormalities in female follicular development.

## MAPK in the physiology of folliculogenesis

### MAPK in primordial follicle formation

Primordial follicle formation is a critical process in ovarian development, serving as the foundation for oocyte maturation and female fertility. It involves the assembly and activation of oocytes encased within GCs, transitioning from a quiescent state to a more active phase. This intricate process is governed by a range of signaling pathways, with the MAPK pathway emerging as a key regulator. Analysis using RNA-seq and ATAC-seq has revealed significant changes in the expression of genes associated with the MAPK signaling pathway during the early ovarian development of geese, particularly during the pre-hatching period when oocyte loss occurs. These alterations suggest that the MAPK signaling pathway may play a crucial role in regulating the formation and assembly of primordial follicles [46]. Furthermore, exposure to DEHP[di(2-ethylhexyl)phthalate] has been shown to result in changes in the miRNA expression profile in mouse ovaries. Some of the differentially expressed miRNAs may regulate ovarian development by targeting mRNAs involved in the MAPK signaling pathway. Gene Ontology (GO) enrichment analysis and pathway enrichment analysis revealed that the target genes of the differentially expressed miRNAs are implicated in the MAPK signaling pathway. This indicates that DEHP may influence the expression of miRNAs, thereby affecting the regulation of MAPK signaling-related genes and subsequently impacting the formation of primordial follicles [47].

Early studies have indicated that the MAPK pathway plays a crucial role in the formation of primordial follicles, primarily functioning as a downstream effector of the KIT(Kinase Insert Domain Receptor) signaling pathway [48]. When KIT ligand (KITL) is added to the culture medium containing mouse fetal or neonatal ovarian tissues, an increase in phosphorylated MAPK (p-MAPK) levels can be observed [48, 49]. Additionally, research has highlighted that KIT can regulate the formation of primordial follicles by activating the PI3K and MAPK3/1 signaling pathways [49]. Gene network analysis in neonatal rats has revealed that ERK1/2 is significantly expressed in several gene modules, correlating with other key genes involved in the assembly of primordial follicles, which suggests that ERK1/2 may regulate follicle assembly by influencing the expression of other genes. Enrichment analysis of gene modules further demonstrated that genes associated with the MAPK signaling pathway are enriched in specific modules, confirming the role of the MAPK signaling pathway in primordial follicle assembly [50]. In adult mice, FSH promotes the assembly of primordial follicles through the FSH receptor 3 (FSHR3), a process that involves the MAPK/ERK pathway [51, 52]. In a study conducted on chickens, FSH and stem cell factor (SCF) were found to enhance ovarian cell proliferation and inhibit apoptosis by activating the MAPK pathway, potentially affecting the assembly of primordial follicles through the regulation of cell adhesion molecule expression [53].

*c-JUN* may participate in the transcriptional regulation during the follicle assembly process [50]. Recently, single-cell level studies have identified two novel genes, *ANXA7* (annexin A7) and *GTF2F1* (general transcription factor IIF subunit 1), that promote primordial follicle formation [54]; notably, *GTF2F1* has been found to interact with JNK in yeast [55]. JNK is specifically localized in oocytes, and its activity increases as germ cell cysts undergo degeneration. The use of the JNK-specific inhibitor SP600125 or knockdown of JNK expression via Lenti-JNK-shRNAs significantly inhibits both the breakdown of germ cell cysts and the formation of primordial follicles [56]. Inhibition of JNK signaling results in abnormal accumulation of E-cadherin between oocytes, suggesting that JNK signaling may regulate the degradation of cysts by modulating E-cadherin expression or function [56]. Furthermore, treatment with Bisphenol S(BPS) leads to abnormal rupture of primordial germ cell cysts and influences subsequent ovarian differentiation while diminishing oocyte quality. The application of JNK inhibitors such as SP600125 can partially counteract the effects of BPS on the rupture of primordial germ cell cysts and the assembly of primordial follicles, further underscoring the important role of JNK signaling in follicle formation [57].

Currently, the role of p38 in the formation of primordial follicles remains underexplored. The activation of the FGF23-FGFR1-p38 MAPK signaling pathway is crucial for maintaining oocyte survival during primordial follicle formation in mice and preventing premature apoptosis [58]. Therefore, further experimental investigations are needed to elucidate the specific functions of the p38 MAPK branch in the formation of primordial follicles and oocyte survival.

### Primordial follicle activation

The role of the MAPK signaling pathway in the activation of primordial follicles is primarily demonstrated through its interaction with the oncoprotein ErbB2. In rat models, *c-erbB2* participates in the regulation of the initiation of primordial follicle growth via the MAPK signaling pathway. Transfection of ovarian tissue with *c-erbB2* siRNA results in a significant downregulation of both *c-erbB2* mRNA and ErbB2 protein levels, accompanied by a marked decrease in the expression of MAPK proteins [59]. Importantly, the changes in p-ERK1/2 protein levels in rat ovaries exhibit a significant positive correlation with *c-erbB2* mRNA expression, indicating a close relationship between the activation state of ERK1/2 and *c-erbB2* expression levels [60]. Epidermal growth factor (EGF) has been shown to promote the development of primordial follicles into secondary follicles through the activation of the MAPK and protein kinase C pathways [61–63]. A plausible mechanism involves the binding of EGF to its receptor EGFR, which may lead to the dimerization of EGFR and ErbB2, promoting the phosphorylation of ErbB2 and activating receptor protein tyrosine kinases (RPTK). The activated RPTK subsequently interacts with proteins containing SH2 domains via its phosphorylated tyrosine residues, activating Ras, which in turn activates Raf, ultimately leading to the activation of ERK1/2. The activated ERK1/2 modulates the activity of transcription factors, thereby influencing gene expression and promoting the initiation of primordial follicle growth [60]. Treatment with PD98059 significantly inhibits the transition of primordial follicles to primary follicles in rat ovarian tissue [62]. In a mouse model where the PTEN(phosphatase and tensin homolog deleted on chromosome ten) inhibitor bpV(HOpic) facilitates primordial follicle activation, U0126 was found to inhibit the phosphorylation of ERK1/2 and follicle activation, further confirming the role of ERK1/2 in follicle activation [64]. Additionally, pentachloronitrobenzene has been shown to enhance the expression of steroidogenic acute regulatory protein(*StAR*) and progesterone production through the activation of the ERK1/2 signaling pathway in rat ovaries, accelerating the transition of primordial follicles to growing follicles [65]. Furthermore, in chicken ovaries, FSH has been reported to activate the MAPK pathway to promote primordial follicle activation, which likely involves multiple factors within the MAPK pathway, such as RAS, RAF, MEK, and ERK [53]. Leukemia inhibitory factor(LIF) and basic fibroblast growth factor (bFGF) significantly enhance the expression of ERK and p-ERK in chicken ovaries, indicating the involvement of the ERK signaling pathway in bFGF-mediated primordial follicle activation [66, 67].

ERK signaling also plays a crucial role in the activation of primordial follicles through the mTOR-KIT pathway. ERK1/2 promotes primordial follicle activation by activating mTORC1 [68]. In murine models, the activation of mTORC1 triggers the recruitment of primordial follicles, with ERK1/2 regulating the activity of mTORC1 during this process [69]. Vasoactive Intestinal Peptide (VIP) treatment has been shown to increase the phosphorylation levels of mTOR and its downstream protein Ribosomal protein S6 (RPS6) in rat ovaries, which aligns with the activation of ERK1/2 [70]. Furthermore, mTORC1 activation leads to the activation of S6K1 and rpS6, resulting in increased expression of KITL (KIT Ligand), thereby facilitating the activation of primordial follicles [64, 69]. Upon binding of KITL to its receptor KIT, the PI3K-Akt signaling pathway is activated in oocytes, promoting the nuclear export of Foxo3a and thus enhancing the activation of primordial follicles [71]. The use of U0126 leads to a decrease in Akt phosphorylation levels and a reduction in Foxo3 nuclear export, indicating that ERK1/2 activity is essential for the activation of the PI3K-Akt signaling pathway [64]. Treatment with U0126 in neonatal mouse ovaries resulted in a significant reduction in the number of activated follicles. U0126 treatment also decreased the phosphorylation levels of Tsc2, S6K1, and rpS6, as well as the expression of KITL, suggesting that ERK1/2 promotes primordial follicle activation via the mTORC1-KITL signaling pathway [64]. Additionally, in *Esr2*-/- rat ovaries, there was an observed increase in ERK pathway activation, which correlates with enhanced primordial follicle activation [72]. The loss of *ESR2* leads to the upregulation of upstream factors such as *KITLG*,* KIT*,* and IGF1*, which can activate RTK, further stimulating the AKT and mTOR pathways [72]. Comparative analysis of embryonic ovarian samples from 17.5 dpc and 15.5 dpc mice revealed a significant upregulation of estrogen receptors and p-ERK1/2 expression, further suggesting that estrogen and the ERK1/2 signaling pathway may be involved in the formation and activation of primordial follicles [73].

**Fig. 1:**
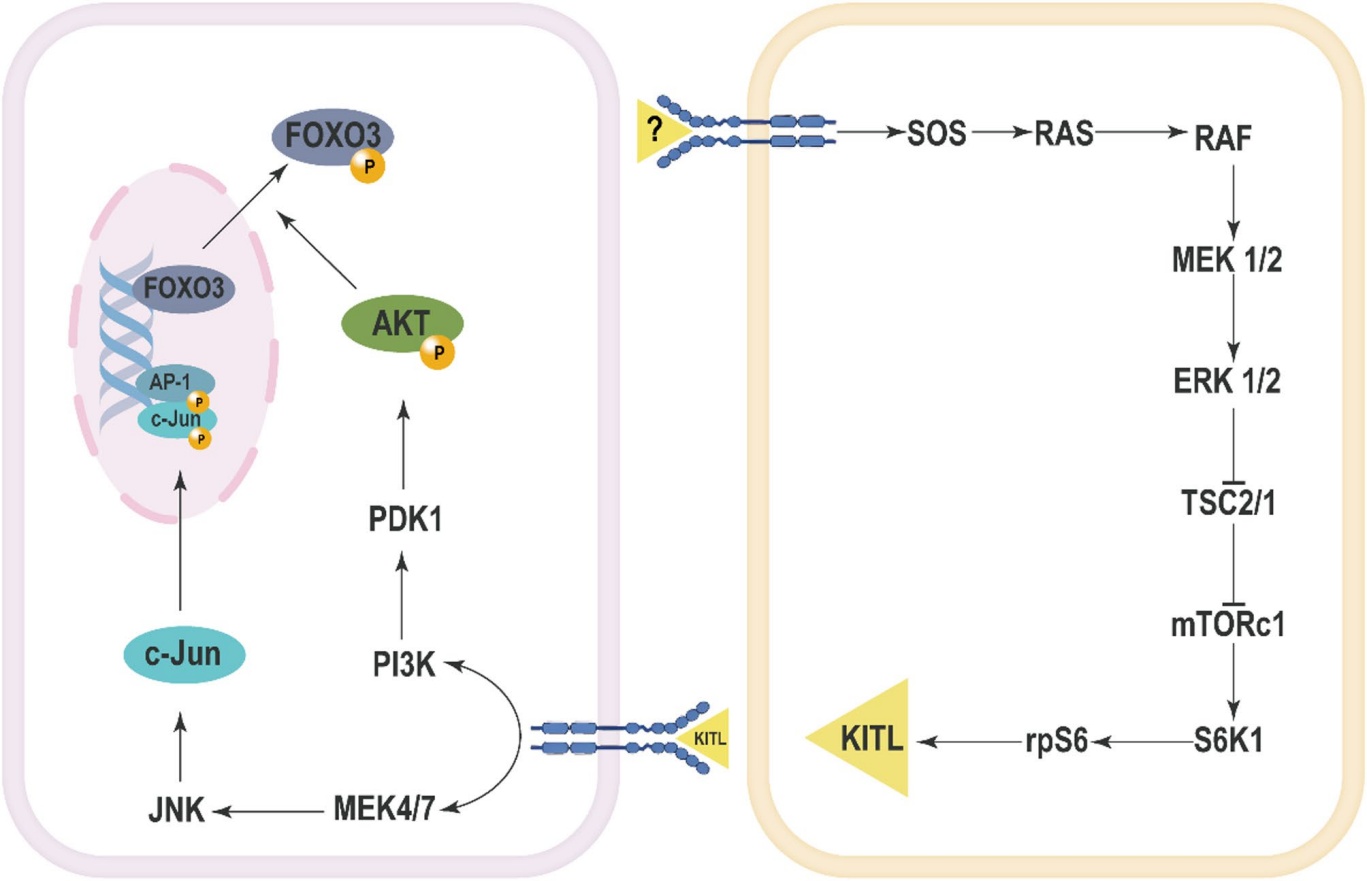
The Mechanisms of activation of primordial follicles involving MAPK pathway

In sheep ovaries, the use of JNK inhibitors, such as SP600125 and JNK inhibitor VIII, significantly impedes the activation of primordial follicles. During the process of follicle activation, Foxo3a is translocated from the oocyte nucleus to the cytoplasm, where it is subsequently degraded following phosphorylation [74]. Inhibition of the JNK pathway can prevent the translocation of Foxo3a from the nucleus, resulting in elevated levels of Foxo3a remaining in the oocyte nucleus, which in turn inhibits the activation of primordial follicles [75]. This suggests that JNK signaling plays a critical role in facilitating the activation of primordial follicles by promoting the nuclear export and degradation of Foxo3a, thereby allowing the process of follicle activation to proceed (Fig. 1).

Recent studies have highlighted the significant roles of MAPK pathway-related non-coding RNAs (ncRNAs) in the activation of primordial follicles. One research team employed a sub-pathway-based approach to analyze the relationship between long non-coding RNAs (lncRNAs) and biological processes during primordial follicle activation. They identified lncRNAs associated with the MAPK signaling pathway, such as FGD5-AS1, which influences primordial follicle activation by regulating genes within the MAPK pathway, including *CRKL*,* HSPA8*,* MAPK8*,* MAX*, and *NFATC3* [76]. Additionally, miR-144-3p plays a crucial role in regulating the p38 MAPK pathway by targeting MAP3K9, significantly impacting both primordial follicle activation and GCs apoptosis [77]. Furthermore, circRNA-miRNA-mRNA network analyses have revealed that circ-009346 and circ-017054 are associated with the MAPK signaling pathway and may participate in primordial follicle activation by influencing this pathway [73]. These findings underscore the intricate regulatory roles of non-coding RNAs in the MAPK signaling pathway and their potential implications in the activation of primordial follicles.

Although the role of MAPK in primordial follicle activation has been initially characterized (Fig. 1), several shortcomings remain in the current studies. Many investigations are primarily conducted in animal models, which may not fully replicate human ovarian physiology. Future research should focus on integrative approaches, including human ovarian tissue studies and advanced molecular techniques, to unravel the complex regulatory networks involved in primordial follicle activation.

### MAPK in steroidogenesis

Current research presents a range of divergent findings regarding the role of the MAPK signaling pathway in steroid hormone production (Table 1) [78–87]. Activation of the MAPK pathway influences the expression of cAMP response element-binding protein (*CREB*), Steroidogenic Factor-1 (*SF-1*), and *DAX-1*, subsequently affecting the expression of *StAR* and steroidogenesis [88, 89].

**Table 1:** MAPK pathway and steroidogenesis

Study (first author; year)	Model system	Gene targeted	When MAPK-related factors are activated
Vijay Simha, Baddela; 2023	bovine primary granulosa cells culture model	ERK1/2, *FOXL2*,* SOX9*,* CYP19A1*,* CYP11A1 and HSD3B*	E2↓, P ↑
Dan, Shan; 2023	rats exposed to lead	JNK, *IRE1α*,* STAR*,* CYP17A1*,* HSD3B1*,* FSHR and CYP19A1*	E2↑, P↓
Biljana, Tesic; 2023	primary cultured human colliculus granulosa cells treated with DEHP	ERK1/2, *CYP19A1 and STAR*	E2↑, P ↑
Liuhui, Li.; 2022	miR-7a2-KO mouse	JNK, *MicroRNA-7a2*	E2↑
Mohammad Reza, Tabandeh; 2022	bovine ovarian granulosa cells treated by LPS	JNK, *3β-HSD*,* CYP19A1*,* FSHR*,* TLR4*,* NLRP3*,* Nrf2*,* NF-Κb*	E2↓, P↓
Bo, Pan; 2022	porcine granulosa cells	ERK1/2, *TIMP3*,* miR-574*	E2↓
Ji-Cheng, Huang; 2022	KGN	JNK, ERK, *CYP19A1*	E2↑
Jung-Chien, Cheng; 2020	primary human granulosa-lutein cells; KGN	ERK1/2, *CYP19A1*	E2↑
Kristina, Pogrmic-Majkic; 2019	human cumulus granulosa cells	ERK1/2, *STAR*,* 3βHSD*,* PPARγ*	P↑
Kristina, Pogrmic-Majkic; 2019	human cumulus granulosa cells	ERK1/2, *STAR*, CYP19A1, *PPARγ*	E2↓, P ↑
Dejun, Xu; 2023	bovine ovarian granulosa cells	Raf, ERK1/2	E2↑, T↑, P↓
Violaine, Simon; 2017	KGN; primary cultures of human luteinized granulosa cells	ERK1/2, *CYP19A1*	E2↓
S, Ebeling; 2011	porcine COCs during in vitro maturation	ERK1/2, *StAR*,* Cyp11A1*,* 3β-HSD*,* Cyp19A1*	E2↓, P ↑
Kenichi, Inagaki; 2009	coculture of rat granulosa cells with oocytes	p38-MAPK, *BMP-2*,* BMP-4*,* Activin*,* FSHR*	E2↑
Tomoko, Miyoshi; 2007	rat primary granulosa cells and coculture with oocytes	ERK1/2, *BMP-6 and BMP-7*	E2↓, P ↑
Nebojsa, Andric; 2006	immature rat granulosa cells	*LHR*,* FSHR*, Aromatase	/
You-Qiang, Su; 2006	mural granulosa cells and cumulus-oocyte complexes from superovulation mice model	ERK1/2, *StAR*,* Cyp11a1*,* Cyp19a1*	E2↓, P ↑
Fu-Qing, Yu; 2005	granulosa cells cultured in vitro from diethylstilbestrol-treated immature rats	p38, *StAR*,* P450arom*,* LRH-1*,* DAX-1*	E2↑, P↓
Fu-Qing, Yu; 2005	granulosa cells cultured in vitro from diethylstilbestrol-treated immature rats	ERK1/2, *StAR*,* PCNA*	E2↑, P ↑
KIMIHISA, TAJIMA; 2003	human granulosa cells; rat preovulatory or preantral granulosa cells	*StAR*,* DAX-1*,* SF-1*	P ↓
R. Kelly, Moore; 2001	primary rat granulosa cells from early antral follicles cultured in serum-free medium	ERK1/2	E2↓, P ↑

*estradiol (E2), testosterone (T), progesterone (P)

Adversely, studies have reported that the activation of ERK1/2 can inhibit estradiol synthesis and/or promote progesterone synthesis [90–92], which may arise from variations in receptor-effector coupling due to differences in cell types, cell line origins, characteristics, and developmental stages. The ERK1/2 signaling pathway has been shown to suppress CYP19A1 expression and estradiol secretion in GCs across various mammalian species. Activated ERK1/2 regulates steroidogenesis in bovine by inhibiting estradiol production while promoting progesterone synthesis, mediated by the downregulation of *FOXL2* and upregulation of *SOX9* [93]. Inhibition of the MAPK pathway within GCs has been associated with increased secretion of 17β-estradiol and decreased secretion of progesterone [90, 92, 94–97]. Specific ERK phosphorylation inhibitors have been shown to upregulate StAR protein expression in human and rat GCs, a regulation that can be blocked by specific PKA inhibitors [91]. In undifferentiated mouse GCs stimulated by FSH, activation of ERK1/2 resulted in increased expression of *aromatase*,* StAR*, and *CYP11A1* [98]. In porcine COCs treated with U0126, an increase in 17β-estradiol secretion and a reduction in progesterone secretion were observed. This treatment also upregulated *Cyp19A1* gene expression while downregulating *3β-HSD* gene expression in porcine COCs[96]. Furthermore, activation of ERK1/2 is critical for the induction of *StAR* mRNA expression and progesterone production in human cumulus GCs by rosiglitazone, with U0126 preventing the rosiglitazone-induced increases in both *StAR* mRNA expression and progesterone production [97].

The JNK pathway plays a significant role in HB-EGF-induced estrogen secretion and apoptosis in KGN cells, primarily mediated through the cAMP-PKA-JNK/ERK-Ca^2+^-FOXO1 pathway [99]. Conversely, other studies indicate that activation of the JNK pathway can impair GCs function by inhibiting steroid hormone synthesis [100].Additionally, in bovine GCs, TNF-α has been shown to inhibit steroidogenesis through the JNK pathway [101].

Studies have shown that FSH can rapidly activate p38 MAPK, and this activation is dependent on protein kinase A (PKA). In rat GCs, treatment with the p38 MAPK inhibitor SB203580 effectively inhibits FSH-induced estradiol production but does not significantly affect progesterone production or cAMP levels [102].

### MAPK in dominant follicle selection

Follicular deviation and the selection of a dominant follicle are critical processes in ovarian physiology, significantly influencing female reproductive outcomes. Follicular bias refers to the preferential development of certain follicles over others, while dominant follicle selection determines which follicle will mature and ovulate. These mechanisms are essential for optimizing oocyte quality and ensuring successful fertilization. Recent studies have highlighted the pivotal role of various signaling pathways in follicular development, with the MAPK pathway emerging as a key regulator. The MAPK pathway mediates crucial cellular processes, including proliferation, differentiation, and apoptosis, thereby influencing follicular dynamics and hormonal responses.

The MAPK signaling pathway, particularly ERK1/2, plays a crucial regulatory role in dominant follicles of cattle, influencing steroid hormone biosynthesis and follicular development [103]. In the early stages of follicular waves in cattle, the levels of phosphorylated MAPK3/1 are significantly elevated in the GCs of follicles that are destined to become dominant [104]. Consistently, phosphorylated Akt and Erk1/2 have also been detected in samples from dominant follicles in sheep, whereas these phosphorylated forms were absent in subordinate follicle samples [105]. On the second day of the follicular wave, the GCs of the future dominant follicle (DF), identified as the largest follicle on that day, exhibited significantly higher levels of p-MAPK3/1 compared to the second largest follicle, suggesting that MAPK3/1 signaling is involved in the formation of the dominant follicle prior to the follicular deviation [106]. After the occurrence of follicular deviation, there were no significant differences in the abundance of p-MAPK3/1 between the GCs of the DF and subordinate follicles (SF), implying that MAPK3/1 may not be essential for the sustained growth of the DF [106]. Furthermore, p-MAPK3/1 levels in the SF did not change significantly by day four, despite undergoing apoptosis, indicating that MAPK3/1 may not participate in the apoptotic process of SF [106]. The TEK signaling pathway, through the activation of the Ras/ERK/MYC signaling cascade, promotes various cellular processes including growth, differentiation, migration, adhesion, proliferation, and survival in dominant follicles [107, 108]. In dominant follicles, the overexpression of *ANGPT1* and *TEK* enhances the activation of the Ras-ERK signaling pathway, which is closely associated with cellular growth and proliferation [107]. *Grb14*, identified as a negative regulator of the MAPK pathway, is highly expressed in SFs, potentially inhibiting MAPK pathway activation, while its low expression in dominant follicles correlates with enhanced MAPK activity, thus facilitating follicle growth and deviation in cattle [109]. Additionally, *TRIB2* expression in dominant follicles is associated with the processes of follicular growth and maturation [110, 111]. *TRIB2* is abundantly expressed in the GCs of dominant follicles and is linked to the activation of the MAPK pathway. Inhibition of *TRIB2* results in reduced phosphorylation levels of ERK1/2 and p38MAPK, alongside increased expression of the cell proliferation marker *PCNA*, indicating that *TRIB2* may negatively regulate GCs proliferation through the MAPK pathway [110]. In summary, the MAPK pathway and its associated factors play a significant role in the processes of follicular deviation or selection, particularly during the early stages of dominant follicle formation. However, once follicular deviation has occurred, the role of MAPK3/1 in maintaining the growth of the DF or the regression of the SF appears to be less significant [106]. In studies utilizing an FSH-induced co-dominant follicle model to assess the effects of FSH on the GCs of the second largest follicles in cattle, despite higher mRNA levels of MAPK1/3 in SF, the abundance of phosphorylated MAPK3/1 proteins in the GCs of control group SF was found to be lower compared to the FSH-treated co-DF2 [112].

During the follicular selection process in cattle, MAPKKK5 (Ask1) may play a critical role in regulating the survival and death of GCs, thereby influencing which follicles are designated as dominant. The activity of the IGF-1 receptor is likely crucial for the maintenance of dominant follicles, as it has the capacity to inhibit apoptosis signals mediated by MAPKKK5 [113]. In yaks, *MAPKAP1* exhibits the lowest expression levels in follicles with diameters ranging from 4 to 6 mm, suggesting that *MAPKAP1* may be associated with follicular development, oocyte maturation, and the selection of dominant follicles [114]. Moreover, *MAPK8IP1* mRNA is expressed in the GCs of dominant follicles and its expression levels increase following hCG treatment in ovulatory follicles. *MAPK8IP1* is known to be associated with the JNK signaling pathway and is involved in regulating processes such as apoptosis, cell motility, adhesion, and morphology. In the context of dominant follicles, *MAPK8IP1* may participate in preventing apoptosis or modulating cellular activity through its effects on cytoskeletal components [115].

### MAPK in COC expansion

**Fig. 2:**
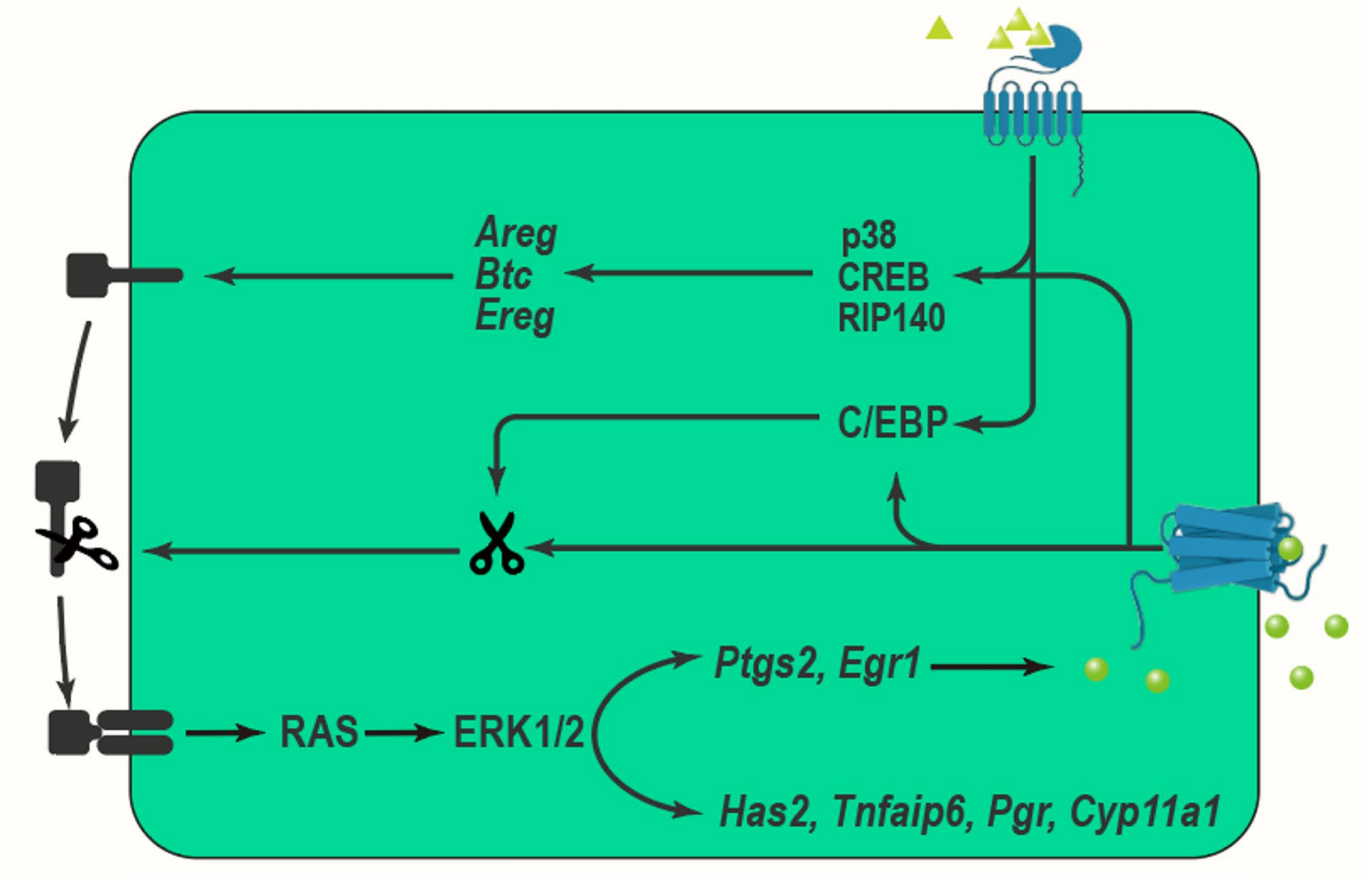
The Mechanisms of COC expansion involving MAPK pathway

The expansion of the COCs, which is a critical process that occurs during the final stages of oocyte maturation and facilitates successful fertilization and subsequent embryonic development, involves the proliferation and differentiation of cumulus cells that secrete hyaluronic acid (HA) and other extracellular matrix components to form a gelatinous matrix surrounding the oocyte. This matrix, which not only protects the oocyte but also enhances its developmental competence, promotes communication between cumulus cells and the oocyte primarily through gap junctions and paracrine signaling. The process, which is tightly regulated by hormonal cues—most notably luteinizing hormone (LH)—triggering the activation of signaling pathways, including the MAPK pathway (Fig. 2), thereby leading to cumulus cell expansion. As this coordinated expansion occurs, it results in the detachment of the COC from the ovarian follicle, ultimately preparing it for ovulation.

LH exerts its effects by binding to its G-coupled receptors, subsequently activating adenylate cyclase and increasing cAMP production. This cascade results in the activation of protein kinase A (PKA) and downstream ERK1/2 signaling pathways [116–118], which play crucial roles in the regulation of EGF-like factor expression [119–121]. LH-induced activation of ERK1/2 leads GCs and cumulus cells to release members of the epidermal growth factor (EGF) family, such as amphiregulin (AREG), epiregulin (EREG), and betacellulin (BTC) [122]. These EGF-like factors then activate the epidermal growth factor receptor (EGFR), which further triggers downstream signaling pathways, including ERK1/2, in both GCs and cumulus cells. This signaling is essential for promoting oocyte meiotic maturation and cumulus expansion [119, 120, 123–125]. Research indicates that while a transient exposure to LH is sufficient to induce oocyte maturation and cumulus expansion, the sustained activity of EGFR is critical for maintaining the prolonged phosphorylation state of ERK1/2 [126, 127].

*RIP140* has been identified as a crucial transcriptional coactivator in the LH signaling pathway. It enhances the transcription of the *Areg* gene by forming complexes with CREB and c-Jun family members. RIP140’s activity at the CRE element on the Areg gene promoter is responsive to forskolin, a compound that activates adenylate cyclase and mimics LH signaling, suggesting its involvement in the activation of the MAPK pathway [128]. EGF-induced phosphorylation of CREB is completely blocked by the ERK1/2 inhibitor U0126, indicating that EGF activates CREB through the ERK1/2 pathway to facilitate cumulus expansion [129]. During the in vitro maturation of porcine oocytes, treatment with EGF/GT1b significantly increases the expression of both total and phosphorylated ERK1/2 proteins [130].

Stimulation by FSH and LH activates the PKA- and p38 MAPK-C/EBP pathways, leading to increased expression of TACE/ADAM17. This enzyme releases the EGF domains of EGF-like factors, subsequently activating the EGFR-ERK1/2-C/EBP pathway, whose activation results in elevated expression levels of TACE/ADAM17 and enhanced *Ptgs2* mRNA expression in GCs and cumulus cells during ovulation [127, 131–134]. TACE/ADAM17, a protease, cleaves EGF-like factors to release their EGF domains, thereby activating the EGFR-ERK1/2 pathway [132, 133, 135, 136]. Using selective inhibitors of TACE/ADAM17 (such as TAPI-2) or siRNA to reduce TACE/ADAM17 activity suppresses luteinization, cumulus expansion, and oocyte maturation in porcine GCs [132]. PGE2 acts on cumulus cells through its receptor PGER2 to maintain cAMP levels, thereby supporting the expression of EGF-like factors and TACE/ADAM17 in a positive feedback loop that is crucial for sustained activation of the ERK1/2 pathway [137–139]. The initial expression of EGF-like factors and TACE/ADAM17 is induced in a gonadotropin-dependent manner, whereas their sustained expression relies on the PGE2-PGER2 pathway [139, 140].

FSH promotes HA synthesis and accumulation in porcine cumulus cells by activating ERK1/2 [141]. Activation of EGFR leads to ERK1/2 activation, which in turn regulates the expression of genes involved in HA synthesis and accumulation [142–144]. When COCs are exposed simultaneously to FSH and IGF1, phosphorylation of AKT and ERK1/2 is significantly increased [145]. IGF1 enhances FSH-induced HA synthesis and retention in the cumulus cell matrix through PI3K/AKT- and ERK1/2-dependent mechanisms [145]. The use of the ERK1/2 inhibitor U0126 markedly reduces HA synthesis and retention stimulated by the combined action of FSH and IGF1 [145], further confirming the critical role of ERK1/2 in cumulus cell expansion [141].

Disruption of ERK1/2 signaling in GCs and cumulus cells underscores the critical role of ERK1/2 activation in mediating LH action during the ovulatory stage of follicular development in vivo [120, 143]. Pharmacological or gene-targeting approaches to downregulate the ERK1/2 pathway result in significant inhibition of COC expansion and oocyte maturation [124]. Inhibition of ERK1/2 significantly reduces the phosphorylation of HDAC2, prevents the deacetylation of H3K27Ac, and disrupts cumulus expansion in mice [146]. Furthermore, inhibition of ERK1/2 activity markedly decreases LH-induced expression of genes associated with COC expansion, such as *Has2*,* Tnfaip6*, and *Ptx3*, which play crucial roles in this process [147]. Treatment with TSA significantly blocks ERK1/2 activation in mouse cumulus cells, indicating that TSA inhibits cumulus expansion by suppressing ERK1/2 activity [148]. Inhibition of the ERK1/2 signaling pathway leads to altered expression of LH-induced genes, including those associated with COC expansion, such as EGR1 and ADAMTS1 [149]. Knockout of ADAMTS1 in mice results in subfertility due to abnormal extracellular matrix remodeling of the follicle wall [149–151].

p38 MAPK differentially regulates the induction of EGF-like factors in cumulus and GCs, with p38 inhibition blocking COC expansion and the expression of specific genes, including *Ptgs2*, which controls PGE2 synthesis. The p38-specific inhibitor SB203580 also inhibits COC expansion and the expression of genes induced by FSH, PGE2, or IL-6 [152–155]. Additionally, SB203580 significantly reduces PGE2-induced expression of AREG, EREG, HAS2, PTGS2, and TNFAIP6, highlighting the importance of p38 in the gene expression associated with cumulus expansion [156]. Treatment with SB203580 also prevents COC expansion in pigs, indicating the crucial role of p38 MAPK in this process [126, 157]. Studies on p38 gene knockout mice reveal that p38 specifically alters gene expression profiles in GCs and COCs. Specific deletion of p38 in GCs leads to upregulation of *Areg* and *Ereg* in GCs but impairs their expression in COCs. This compensatory upregulation in GCs may help maintain the fertility of p38 GC-/- mice. Despite impaired responsiveness of COCs to FSH, forskolin, or PGE2 under in vitro conditions, co-culture with AREG allows normal COC expansion, suggesting that GC-derived AREG and EREG can bypass the requirement for p38 signaling in PGE2-induced *Areg/Ereg* expression in COCs, thus maintaining COC expansion [158], which implies that while p38 is not absolutely essential for pre-ovulatory follicle development or ovulation and luteinization-related events in vivo, it does impact gene expression. Moreover, treatment with traditional Chinese medicine components BSTJ-II-D and BSTJ-III-D increases serum FSH levels and the expression of p-p38/p38 and p-ERK1/2/ERK1/2 proteins in mice, suggesting that these compounds may promote cumulus cell expansion by regulating FSH levels and activating the MAPK signaling pathway [159].

### MAPK in ovulation

Ovulation, which represents a pivotal event in the female reproductive cycle, involves the release of a mature oocyte from the ovarian follicle, a process that is intricately regulated by a cascade of hormonal signals. This culminates in the surge of LH, which not only induces final oocyte maturation but also triggers a series of biochemical changes within the follicle, resulting in the weakening of the follicular wall. As the follicle ruptures, the oocyte, surrounded by cumulus cells, is expelled into the peritoneal cavity.

LH activates RAS through its receptor, and the activation of RAS induces ERK1/2 phosphorylation via a receptor tyrosine kinase-dependent mechanism, which is crucial for ovulation [119, 120, 142, 160, 161]. During the ovulatory process, *Areg* and *Ereg* are expressed in GCs and cumulus cells [119, 121], with their induction accompanied by increased levels of *TACE/ADAM17* mRNA [132, 134, 138]. The use of the ERK1/2 pathway inhibitor PD0325901 completely inhibits ovulation in mice, resulting in anovulation, a phenotype similar to that observed in mice with GCs-specific *ERK1/2* gene knockout (KO) [120]. In mouse ovaries, KRAS is highly expressed in GCs of growing follicles, and active RAS and phosphorylated ERK1/2 levels significantly increase following hCG treatment [120, 162]. In rats, ERK plays a critical role in ovulation by regulating the expression of *UGT2B15* [163].

Sustained ERK1/2 signaling is essential for follicle rupture during ovulation. When PD0325901, an ERK1/2 pathway inhibitor, is administered 4 h after hCG stimulation in mice, follicle rupture is adversely affected [164]. LH/hCG activates EGF-like growth factors, which in turn activate COX-2, thereby increasing the biosynthesis of PGE2. PGE2, through its receptors EP2 and EP4, further induces the expression of AREG and EREG, which also activate ERK1/2, creating a positive feedback loop [119, 120, 165]. Additionally, PGE2 promotes cumulus cell expansion through its receptors EP2 and EP4, a critical step preceding ovulation [166].

During the follicle development process prior to ovulation, genes induced by FSH or eCG and typically downregulated by LH (including *Fshr*, *Lhcgr*, and *Nr5a2*) remain elevated in *ERK1/2* GC-/- mice. Conversely, the expression of genes associated with cumulus expansion and ovulation (such as *Has2*,* Tnfaip6*,* Ptgs2*,* Ptx3*, and *Pgr*) is entirely absent in these *ERK1/2* GC-/- mice, which indicates that ERK1/2 is essential for terminating the expression of genes controlling GCs proliferation and for inducing genes that regulate luteinization and ovulation [167]. CD24, by activating the EGFR-ERK1/2 pathway, increases the expression of prostaglandin synthesis enzymes (such as *AKR1C1*,* PTGS2*,* PTGES*,* PLA2G4A*) and prostaglandin transport proteins (such as *SLCO2A1* and *ABCC4*), which play roles in ovulation and luteinization [168]. Inhibiting ERK1/2 activity significantly reduces LH-induced *Egr1* mRNA and protein expression, with *Egr1* being a critical transcription factor regulating ovulation-related genes such as *Ptgs2* [147]. A detailed study on the roles of C/EBPα and C/EBPβ in regulating ovulation events demonstrates that these transcription factors are crucial for follicle rupture and luteinization, but *Cebpa/b* gc-/- GCs-specific knockout mice do not show significant defects in LH-induced oocyte maturation and cumulus expansion [169]. By comparing the transcriptomes of GCs from *Cebpa/b* gc-/- and *Erk1/2* gc-/- mice 4 h after hCG injection, it was found that only a subset of genes regulated by Erk1/2 (approximately 19%) are controlled by C/EBPα/β [169]. This suggests that other transcription factors mediate ERK1/2’s role in pre-ovulatory follicles. EGR1 is a prime candidate as its expression is suppressed in mice with disrupted ERK1/2 signaling, and silencing *Egr1* in GCs reduces *Ptgs2* expression levels, a key gene involved in follicle rupture [147]. Additionally, *LRH1* (liver receptor homolog-1, also known as NR5A2), a transcription factor phosphorylated and functionally activated by ERK1/2 [170], is a potential target of RAS-ERK1/2 in GCs of pre-ovulatory follicles [170, 171]. LRH1, structurally similar to steroidogenic factor 1 (SF1/NR5A1), is expressed in GCs of growing follicles. Recent studies indicate that LRH1 is a potential RAS-ERK1/2 target in pre-ovulatory follicular GCs. Conditional disruption of the *Lhr1* gene in GCs results in ovulation and luteinization defects in mutant mice [171], whereas conditional disruption of the *Nr5a1* gene in GCs selectively impairs early follicle growth rather than the ovulation stage [172].

The p38 MAPK signaling pathway plays a significant role in regulating the expression of *Nts*, which is considered a key regulator in the ovulation process. Therefore, it can be inferred that the p38 MAPK signaling pathway indirectly affects ovulation by modulating *Nts* expression [173]. During ovulation, the MAPK signaling pathway promotes the inflammatory response by activating downstream transcription factors such as *AP-1*. This involves the production of pro-inflammatory cytokines, which are essential for various aspects of the inflammatory response, including vasodilation, leukocyte mobilization, and tissue remodeling. For instance, the activation of FOS and JUN can aid in attracting leukocytes to the ovulation site by promoting the expression of inflammatory cytokines and chemokines [174]. In *Prdx1* knockout mice, the absence of PRDX1 leads to increased activation of the ERK1/2 and p38 signaling pathways, whose heightened activation is potentially associated with increased ROS production and apoptosis in COCs after ovulation [175].

### MAPK in luteinization

Luteinization, the process by which GCs and TCs transform into luteal cells following ovulation, involves cell proliferation and differentiation that require the action of LH and the activation of the MAPK signaling pathway. The activation of ERK1/2 is crucial during the luteinization phase of follicle development triggered by LH during ovulation [120, 142, 143]. LH exerts its effects by binding to its receptor (LHCGR), which activates protein kinase A (PKA) and subsequently enhances the phosphorylation and expression of MAP3K8. This, in turn, facilitates the phosphorylation of ERK1/2 and promotes the expression of target genes, ultimately aiding in progesterone synthesis [169, 176, 177]. Expression of MAP3K8 is observed in mouse luteal cells, with elevated levels noted during mid-luteal development. Inhibition of MAP3K8, using both MAP3K8 siRNA and specific inhibitors, significantly impairs progesterone synthesis [178]. Additionally, E2 increases MAP3K8 expression via the GPR30 receptor, enhancing ERK1/2 phosphorylation and stimulating progesterone synthesis in mice [178]. Following MAP3K8 inhibition, the expression of transcription factors such as *DAX1* and *FOXL* is upregulated, suggesting they may act to suppress the expression of steroidogenic genes in porcine luteal cells [176].

ERK1/2 acts as an intermediary in the intracellular signaling pathway stimulated by LH inGCs, regulating the expression of multiple genes, including *StAR* and *cyp19a1* [179]. The activation of the *StAR* gene is critical for progesterone synthesis [120]. Studies in mice indicate that a deficiency in ERK1/2 signaling leads to reduced *STAR* expression and decreased circulating progesterone levels [95, 149]. Additionally, two genes associated with histone modification and chromatin remodeling, NCOA7 and HP1BP3, function downstream of the ERK-1/2 signaling pathway and are involved in the luteinization of GCs following ovulation [179]. The activation of ERK-1/2 signaling leads to the recruitment of the CBP/p300 histone acetyltransferase, which participates in the acetylation of histones within the promoter regions of LH-induced target genes [179, 180]. Moreover, the activation of ERK1/2 results in the expression of downstream genes such as *Bhmt* and *Ptgfr*, which play significant roles in luteal maintenance and steroidogenesis [181]. LH-induced ERK1/2 activation also stimulates the transcription factors CCAAT/enhancer-binding proteins α/β (C/EBPα/β), which are involved in the luteinization process, including new blood vessel formation [169, 182]. Notably, the expression of C/EBPβ decreases following MAP3K8 inhibition, aligning with the downstream regulatory effects of the ERK1/2 signaling pathway [176].

Kisspeptin treatment enhances the transcription levels of key steroidogenic enzymes, including StAR, CYP11A, and 3β-HSD, by stimulating the ERK1/2 signaling pathway in rat luteal cells, which may facilitate luteal formation and support the progression of pregnancy [183]. In a mouse model with *Lgr4* gene knockout, the absence of the *Lgr4* gene led to a decreased activity of the EGFR-ERK signaling pathway in luteal cells, ultimately affecting luteal formation and function. Notably, HB-EGF was able to restore the expression of luteal marker genes in GCs from *Lgr4* knockout mice [181]. Furthermore, in primary cultures of human luteal GCs, HB-EGF can activate ERK1/2 via EGFR and HER4 receptors, thereby promoting *StAR* expression and progesterone production [184]. Additionally, progesterone secretion in mice is enhanced by the phosphorylation of ERK1/2 induced by POP. Inhibition of the ERK signaling pathway using U0126-EtOH in mouse luteal cells resulted in reduced progesterone production, along with decreased expression of steroidogenic enzymes and SF1 [185]. SF1 is a transcription factor crucial for progesterone synthesis. Overexpression of POP increased SF1 expression, whereas inhibition of the ERK signaling pathway decreased SF1 levels, indicating that the ERK pathway influences progesterone synthesis by regulating SF1 expression [185]. Moreover, EG-VEGF/PK-1 promotes the proliferation of luteal endothelial cells through the activation of ERK1/2 [186, 187].

In human luteal GCs, IL-1β activates the ERK1/2 and p38 pathways, and inhibition of these pathways attenuates IL-1β-induced CREB phosphorylation, StAR expression, and progesterone synthesis [188]. In vitro studies using specific MAPK14 inhibitors such as SB203580 on canine luteal cells revealed significantly decreased concentrations of P4 and E2 under insulin stimulation, indicating that MAPK14 plays a crucial role in insulin-induced steroidogenesis [189]. Furthermore, treatment with hCG and LH activates the JNK pathway, thereby enhancing the steroidogenic capacity of human luteal GCs and increasing progesterone production. This activation is accompanied by upregulation of steroidogenic enzymes StAR and 3β-HSD [190]. Conversely, Activin-A has been shown to decrease JNK activity, leading to reduced expression of steroidogenic enzymes such as StAR and 3β-HSD, ultimately resulting in decreased production of E2 and P4 in human luteal GCs [191].

The role of the MAPK pathway in steroidogenesis may vary depending on cell type and physiological conditions. In bovine follicles with inhibited ERK1/2, the expression levels of StAR protein in GCs and TCs increased, suggesting that ERK1/2 may influence the luteinization process through different mechanisms [149]. LiCl rapidly increases ERK1/2 phosphorylation and subsequently downregulates StAR in human luteal GCs [192]. Growth differentiation factor-8 (GDF-8) activates the ERK1/2 signaling pathway, which correlates with the downregulation of StAR expression and reduced progesterone production, potentially playing a significant role in preventing premature luteinization before ovulation and maintaining normal follicular development [193]. Moreover, the relationship between PGF2α, luteinization, and luteal regression remains poorly understood. PGF2α activates phospholipase C via its Gq-coupled receptors on target cells, leading to increased intracellular calcium and enhanced PKC activity, which subsequently activates MAPK signaling cascades, including ERK, p38 MAPK, and JNK [194–198]. PGF2α activates ERK in luteal endothelial cells and rapidly stimulates the expression of immediate early response genes (such as *c-fos* and *c-jun* mRNA), promoting luteal cell proliferation [199]. Conversely, the activation of MAPK-associated kinases is linked to the inhibition of steroid synthesis during luteal regression [197, 198, 200]. ERK signaling is activated by PGF2α in luteal cells from various species, including rats, pigs, cows, and humans [198, 201, 202]. PGF2α induces early growth response 1 (EGR1) gene expression via the RAF/MEK1/ERK signaling pathway, which leads to increased expression of transforming growth factor β1 (TGF-β1) associated with luteal regression in cattle [203]. TGF-β1 activates the ERK1/2 signaling pathway, which participates in TGF-β1-induced aromatase expression and E2 production. Pre-treatment with U0126 in KGN cells diminished TGF-β1-induced aromatase mRNA and protein levels [81]. The activation of ERK1/2 is essential for autophagy during PGF2α-induced luteal regression in rats, independent of mTOR regulation [204]. In PGF2α-treated rat luteal cells, the activation of ERK signaling correlates with increased expression of *ATF3* and decreased progesterone concentrations [198]. JNK activation is associated with apoptotic cell death during luteal regression in buffalo [195]. In PGF2α-treated rat luteal cells, JNK activation correlates with increased *MCP-1* mRNA expression [205]. PGF treatment elevates *ATF3* expression in rat luteal tissue, while heat stress decreases PGF-induced ATF3 expression [200]. The activation of JNK precedes the increase in ATF3 expression under PGF2α treatment, suggesting that JNK may play a role in the regression of buffalo luteal tissue [197]. Furthermore, PGF2α decreases progesterone levels by inhibiting CK1α expression, while simultaneously activating ERK and SP1, thereby promoting the expression of *20α-HSD* and the production of *20α-OHP*, which may be a key mechanism in promoting parturition at the end of pregnancy [206, 207].

RAS proteins also play a significant role in the formation and regression of the corpus luteum, primarily by regulating signaling pathways associated with angiogenesis and apoptosis. In bovine luteal cells, the expression of RASAL3 and RASA3 proteins significantly increases during the secretory phase compared to the proliferative phase, whereas RasGEF1B levels decrease. The expressions of vascular endothelial growth factor A (VEGFA), VEGF receptor 2 (VEGFR2), and angiopoietin 1 (Ang1) significantly rise during both the proliferative and secretory phases, consistent with the regulatory role of RAS proteins. Analysis using the STRING database identified that RasGEF1B is activated via estrogen receptor α (ERα) and subsequently activates H-Ras and R-Ras through VEGFA and VEGFR2 [208]. VEGFR2 plays a critical role in the proliferation, migration, and tubular structure formation of endothelial cells. The MEK/ERK signaling pathway mediates the downregulation of VEGFR2 expression in mice, which is associated with the anti-angiogenic effects induced by microcystin-LR (MC-LR). The use of p-ERK agonists can mitigate the anti-angiogenic effects of MC-LR, indicating that the MEK/ERK signaling pathway is crucial for the construction of the luteal vascular network. SP1 (specificity protein 1), as a transcription factor, is involved in the transcriptional regulation of VEGFR2, and the MEK/ERK/SP1 signaling pathway is inhibited by MC-LR [209]. In cultured mouse luteal cells, pre-treatment with the ERK inhibitor PD98059 can suppress PGF2α-induced SP1 expression and completely block the enhancement of SP1 activity stimulated by PGF2α [206].

In summary, these studies indicate that the activation of MAPK is essential for GCs luteinization and luteal regression [94, 189, 210]. However, further evidence is needed to elucidate the specific roles of the MAPK pathway during different stages and under various stimuli in the process of luteinization. Understanding these dynamics will provide deeper insights into the regulatory mechanisms governing ovarian function and may lead to improved therapeutic strategies for disorders related to luteal phase deficiencies.

### MAPK in ovarian cell death

**Table 2:** MAPK pathway and ovarian cell death

Study (first author; year)	Model system	Gene targeted	When MAPK-related factors are activated
Lingcong, Deng; 2024	porcine ovarian granulosa cells	ERK1/2, p38, JNK, Bax, caspase 9	apoptosis ↑
Ruochen, Yang; 2023	ovine ovarian granulosa cells	MAPK12, *L-PRLR*,* S-PRLR*	apoptosis ↑
Xiaohong, Han; 2023	granulosa cells of yaks	JNK, *LncRNA MEG3, miR-23a*, ASK1	apoptosis ↑ autophagy ↓
Yuxi, Liu; 2023	POF mice induced by cyclophosphamide; isolated and cultured mouse granulosa cells	ERK1/2, NEAT1, *miR-654*, STC2	apoptosis ↑ autophagy ↑
Guangfa, Zhang; 2022	chicken granulosa cells in vitro	*miR-122-5p* and MAPK3	apoptosis ↓
Jiaxin, Duan; 2021	porcine COCs	MEK1/2, ERK1/2, Connexin 43 (Cx43)	autophagy ?
Muhammad, Safdar; 2021	mouse primary granulosa cells	ERK1/2, orexin receptor type 1	apoptosis ↓
Q.E., Xie; 2021	chinese hamster ovary	ERK, P38, LC3	autophagy ?
Xing-Yu, Zhou; 2021	KGN; rat	JNK, p38, p21	proliferation ↓
Meimei, Wang; 2023	bovine granulosa cells	ERK1/2, p38, NRF2,BCL2, BAX, CASP3, CCND1, P21	ERK1/2: apoptosis ↓ p38: apoptosis ↑
Junyan, Sun; 2021	chronic unpredictable stress (CUS) mouse model; KGN	ERK, JNK, p38, Isocitrate dehydrogenase-1	autophagy ↑
Xingde, Du; 2021	mice treated by Microcystin-LR; KK cell line	JNK, p38, P53, Fas, MKK4, MKK3, *Ddit3*, *Mef2c*	apoptosis ↑
Mingquan, Huang; 2020	KGN	JNK, Bax, Bak, Bid, Bcl-2, Bcl-xL, *Mcl-1*	apoptosis ↑
Mingquan, Huang; 2021	KGN	JNK, Bax, Bak, Bcl-2,Mcl-1, Caspase-3, ASK1	apoptosis ↑
Peijie, Wang; 2020	goat granulosa cells	ERK1/2, PTX3	apoptosis ↓
Shuo, Liu; 2019	porcine ovarian granulosa cells	JNK, protein kinase C delta	apoptosis ↑
Cai-Hong, Zhang; 2018	HTR-8/Svneo; non-pregnant ICR mice	ERK1/2, BRAF, MITF, RAS	apoptosis ↓
Fenglei, Chen; 2018	mouse ovarian granulosa cells	ERK1/2, CREBZF	apoptosis ↓
Yi-Ru, Wang; 2018	bovine ovarian granulosa cells under heat stress	ERK1/2, Heme oxygenase 1	apoptosis ↑
Sun-Ji, Park; 2018	*prx2*-deficient mice and mouse; embryonic fibroblasts (MEFs) derived from these mice	JNK, *Peroxiredoxin 2 (Prx2)*	apoptosis ↑
Yan, Cao; 2018	mouse granulosa cells	JNK, BCL-2, BECN1	autophagy ↑
Mei-Jou, Chen; 2017	mouse nonluteinized granulosa cells; human ovarian tissue sections	p38, *p53*,* p21*	proliferation ↓
Ying, Han; 2017	human granulosa cells; COV434	ERK1/2, SIRT1	apoptosis ↓
Hongyan, Yang; 2017	COV434	JNK, p53, Puma	apoptosis ↑
Qiannan, Weng; 2016	mouse follicular granulosa cells	JNK and FoxO1	apoptosis ↑
Hui, Gao; 2016	porcine granulosa cells	JNK, NF-kB, LC3, beclin1	autophagy ↑
Yanqing, Geng; 2014	rats and primary ovarian granulosa cells	ERK, JNK, Bcl-2, Bax, Caspase-3	ERK, JNK: apoptosis ↑
Indrajit, Chowdhury; 2013	undifferentiated rat granulosa cells	ERK1/2, Prohibitin	apoptosis ↓
Melike, Sapmaz-Metin; 2013	rat ovaries subjected to torsion-detorsion injury	JNK, p38	apoptosis ↑
Sakhila K., Banu; 2011	primary cultures of rat granulosa cells	ERK1/2, Bcl-2, Bcl-XL, BAX, BAD, HSP70 HSP90, p53	apoptosis ↑
A, Rak-Mardyla; 2010	prepubertal porcine ovaries and ovarian follicular cells	ERK1/2, GHSR-1a	apoptosis ↓
Justin H, Turner; 2006	CHO-K1 cells expressing human 5-HT1A receptor (CHO-5-HT1AR cells)	ERK1/2, JNK, 5-HT1A receptor	ERK1/2: apoptosis ↓ JNK: apoptosis ↑
André, Tanel; 2006	chinese hamster ovary cells	ERK1/2, p38, JNK, AKT, PKB, p53	ERK1/2, p38: apoptosis ↑ JNK:?
J., Uma; 2003	macaca radiata	JNK, p38,Bax, Caspase-2, Caspase-3	apoptosis ↑
Masayuki, Shiota; 2003	porcine granulosa cells	ERK1/2	ERK1/2: apoptosis ↓ p38: apoptosis ↑
J.J., Peluso; 2003	spontaneously immortalizedgranulosa cells; immature rat ovaries	ERK1/2, Progesterone Binding Protein (P4BP)	apoptosis ↑
Xiaoming, Hu; 2002	rats treated by 4-vinylcyclohexene diepoxide (VCD)	JNK, p38, AP-1	apoptosis ↑
Shah M., Khan; 2000	human luteinized granulosa cells	Raf-1	apoptosis ↓
Gerd, Gebauer; 1999	granulosa cells from 21-day-old immature female rats treated with eCG (equine placental prolactin)	Raf-1, Bad, Bax, Bcl-2	apoptosis ↓
Rush H., Oliver; 1999	human luteinized granulosa cells from in vitro fertilization aspirates	ERK1/2	apoptosis ↓

#### Apoptosis

The ERK signaling pathway plays a critical role in cell proliferation and survival, and its inhibition can lead to apoptosis and dysfunction of ovarian cells (Table 2) [211–217]. In chickens, *miR-122-5p* can inhibit GCs proliferation and promote apoptosis by targeting MAPK3. Knockdown of MAPK3 results in significantly decreased proliferation and reduced numbers of EdU-positive cells. *MiR-29* activates the ERK1/2 signaling pathway by targeting *PTX3*, leading to an increased ratio of Bcl2 to Bax and a significant downregulation of caspase-3 protein expression, which inhibits apoptosis in goat GCs [218]. When ERK activity is inhibited by PD98059 in GCs isolated from prepubertal pigs, serum or FSH-induced cell survival is suppressed [219]. Co-treatment with ghrelin and PD98059 significantly reduces proliferation and increases apoptosis in bovine GCs [220]. Raf-1, an upstream activator of the ERK signaling pathway, shows decreased activity in line with reduced ERK activity, suggesting its potential role in apoptosis [221]. Depletion of *Raf-1* using geldanamycin results in lowered MAP kinase activity, activating caspase-3 and ultimately leading to apoptosis in human luteinized GCs [222]. Activation of the MAPK pathway is essential for the survival of human luteinized GCs, as treatment with PD98059 increases the percentage of subdiploid apoptotic cell [223]. Interestingly, activation of the ERK pathway can also exacerbate apoptosis in GCs under certain conditions (Table 2) [224–228].

Generally, p38 and JNK primarily play a role in promoting apoptosis during follicular development, particularly under stress conditions (Table 2) [229–232]. As an upstream regulator of the p38/JNK pathway, ASK1 is closely associated with apoptosis and organ injury. Radiation exposure markedly upregulates the levels of TGF-β protein in the ovaries of rats, subsequently activating downstream MAPKs, including p38 and JNK [233].

p38 exhibits dual roles in cell survival and apoptosis, which are contingent upon the type of stimuli encountered (Table 2) [211, 219, 234–243]. Activation of JNK promotes the nuclear translocation of FoxO1, thereby influencing the apoptosis process [241]. Studies have shown that ovaries from *Prx2−/−* mice exhibit more apoptotic cells at 18 months of age, along with elevated levels of apoptotic-related proteins such as Bax, cytochrome c, cleaved caspase-3, and p-JNK [244]. Furthermore, JNK activation has been linked to CrVI-induced apoptosis. CrVI increases JNK phosphorylation, which may promote the translocation of Bax protein to mitochondria, thereby facilitating apoptosis [245]. Interestingly, JNK appears to play a role in the survival pathway against acetaldehyde-induced damage. Following treatment with the JNK inhibitor SP600125, the cell death mode induced by acetaldehyde in Chinese hamster GCs shifts from apoptosis to necrosis [227].

#### Autophagy

Autophagy is a process of self-digestion in cells that can clear damaged or dysfunctional organelles and promote repair when occurring at moderate levels. Overexpression of NEAT1 can indirectly increase the expression of STC2 by reducing *miR-654* levels, subsequently decreasing apoptosis and autophagy in mouse GCs via the STC2/MAPK pathway (by lowering p-ERK1/2 levels) [246]. Additionally, activation of ERK1/2 is also involved in the activation of autophagy induced by oxidative stress in KGN cells [247]. In Chinese hamster ovarian cells, overexpression of LC3 can activate ERK and p38 signaling pathways, while melatonin treatment can restore this activation state [248]. Furthermore, melatonin inhibits autophagy by maintaining the interaction between BCL-2 and BECN1, suggesting that melatonin may prevent oxidative stress-induced autophagic cell death via the JNK/BCL-2/BECN1 signaling axis [249]. 17β-estradiol has been shown to inhibit the MEK/ERK signaling pathway through autophagy-mediated mechanisms, affecting the phosphorylation of gap junction intercellular communication and Cx43, thereby influencing nuclear maturation of porcine COCs [250]. Treatment of porcine GCs with FSH or the NF-kB inhibitor PDTC resulted in the activation of the JNK signaling pathway, as well as increased expression levels of autophagy markers LC3-II and Beclin1, indicating that FSH or PDTC enhances autophagic activity through the JNK signaling pathway [251].

Currently, the role of MAPK in apoptosis has been well characterized (Table 2); however, the causal relationship and the sequence of activation between the MAPK pathway and the occurrence of autophagy remain unresolved mysteries. Promisingly, emerging studies investigating the relationship between novel forms of cell death and the MAPK pathway present significant research opportunities.

## MAPKs conditional regulation of ovarian physiology: effects on female reproductive health

### MAPK in ovarian aging

Ovarian aging involves the gradual decline of follicle reserves and hormonal function, characterized by alterations in MAPK signaling pathways. As age increases, oxidative stress elevates, activating pathways like JNK and p38, leading to cellular dysfunction and apoptosis in GCs.

Gene expression profiling indicates that genes associated with the MAPK pathway exhibit significant alterations in the ovaries of aged macaques and sows [252, 253]. Notably, differences in MAPK1 expression levels were observed between GCs from aged and young non-human primates, with LASSO regression analysis identifying MAPK1 as an independent factor capable of distinguishing ovarian aging [254]. Activation of the ERK pathway may lead to premature activation of primordial follicles and deplete follicle reserves, thereby accelerating ovarian aging [72]. Additionally, in GCs from Kunming mice undergoing reproductive aging, MAPK1 expression levels were upregulated, further supporting the potential role of MAPK1 in ovarian senescence [254]. In a menadione-induced oxidative stress model, KGN cells showed increased MAPK1 expression, and the addition of the antioxidant N-Acetyl Cysteine(NAC) did not reverse this upregulation, suggesting that MAPK1 acts as a regulatory factor in the oxidative stress response rather than a downstream effector [72]. Furthermore, FSH operates through its alternative splice variant FSHR3 in the ovarian surface epithelium (OSE), facilitating calcium signaling and activating the MAPK/ERK pathway to promote stem cell proliferation [51, 255, 256]. As age increases, the microenvironment may become impaired, hindering normal stem cell differentiation and contributing to ovarian aging [51, 257, 258]. Under the influence of chronic unpredictable stress (CUS), there is a decline in IDH1(Isocitrate dehydrogenase 1) expression, leading to reduced antioxidant capacity, which subsequently enhances oxidative stress and activates MAPK signaling pathways, including ERK, JNK, and p38. Among these, ERK is associated with autophagy activation, while JNK and p38 are linked to cell cycle arrest. These biological alterations result in dysfunction of GCs, thereby promoting ovarian aging [247].

The role of the JNK pathway in ovarian aging is mediated through the regulation of oxidative stress responses and the impact on steroidogenesis [259]. ASK1 is activated in response to cellular oxidative stress damage, subsequently activating JNK to induce downstream signaling [260]. In mice, Propylparaben(PrPB) exposure induces the oxidative stress-related JNK pathway, leading to the accumulation of oxidative damage products, activation of antioxidant responses, mitochondrial dysfunction, and impaired ATP and steroid production by GCs. Melatonin treatment can partially reverse the steroidogenic dysfunction induced by PrPB by inhibiting the JNK signaling pathway in GCs [259]. In ovariectomized (OVX) aging rats, protein levels of p-ASK1, p-MKK7, p-JNK, p-c-Jun, and CASP3 were significantly elevated compared to the sham-operated control group with intact ovaries. Treatment with the Tiaogeng Decoction restored the expression levels of these proteins to those observed in healthy sham-operated controls [261]. Gel Y + Inh significantly inhibited p-JNK signaling, thereby regulating cellular function and influencing ovarian aging in rats [262]. In *Prx2*-/- mice, the ability to scavenge ROS in the ovaries was diminished, leading to increased ROS levels and subsequent activation of the JNK pathway. Activated JNK promotes the expression of pro-apoptotic proteins, such as Bax, cytochrome c, and cleaved caspase-3, resulting in enhanced ovarian cell apoptosis [244]. The He’s Yangchao Recipe may counteract the age-related decline in ovarian function in mice by inhibiting the expression of JNK and p53, thereby reducing oxidative stress [263].

Aging elevates the basal level of p38 phosphorylation in human GCs. Interestingly, the subcellular localization of activated p38 is distinctive: it primarily resides in the nucleus of young cells and in the cytoplasm of aged cells. Treatment of H2O2-treated KGN cells with NAC and the p38 inhibitor SB203580 can block the activation and cytoplasmic localization of p38 MAPK [264]. Oxidative stress may induce the activation of the MAPK family; in aged human GCs, the expression of the aging marker *GSTT1*(Glutathione S-Transferase Theta 1) is upregulated, consistent with the activation of p38 MAPK [264]. In mouse GCs, inhibitors of JNK and p38 significantly reduce the proportion of cells in the S phase and G2/M phase caused by IDH1 downregulation while increasing the levels of cell cycle-related proteins CCNB1 and CDK1. The ROS scavenger NAC can inhibit the activation of MAPK signaling pathways induced by IDH1 downregulation, thereby reducing cellular aging and cell cycle arrest [247]. In oocytes from aging female mice, decreased GST activity and thiol levels indicate that these oocytes may be subjected to oxidative stress, with the MAPK pathway, particularly the pro-apoptotic JNK/SAPKs and p38 MAPK family, potentially involved in this process [265], which further supports the role of oxidative stress in ovarian aging.

In human ovarian surface epithelial cells (HOSEpiCs), the inhibition of YTHDF2 (YTH N6-Methyladenosine RNA Binding Protein F2) leads to increased mRNA stability of *MAP2K4* and *MAP4K4*, which may promote the expression of the senescence-associated secretory phenotype (SASP) through the activation of the NF-κB signaling pathway, which is associated with inflammation and cellular dysfunction during ovarian aging [266]. Melatonin may counteract ovarian aging by restoring YTHDF2 expression, which indirectly affects the expression of MAP2K4 and MAP4K4, thereby inhibiting the activation of the NF-κB signaling pathway and the expression of SASP [266]. Additionally, studies have shown that gene expression related to the MAPK cascade changes following mesenchymal stem cell (MSC) transplantation. Notably, the expression level of the *Map4k1* gene is significantly elevated, indicating that the MAPK pathway may play a crucial role in MSC-mediated therapy for ovarian aging in mice [267].

**Fig. 3:**
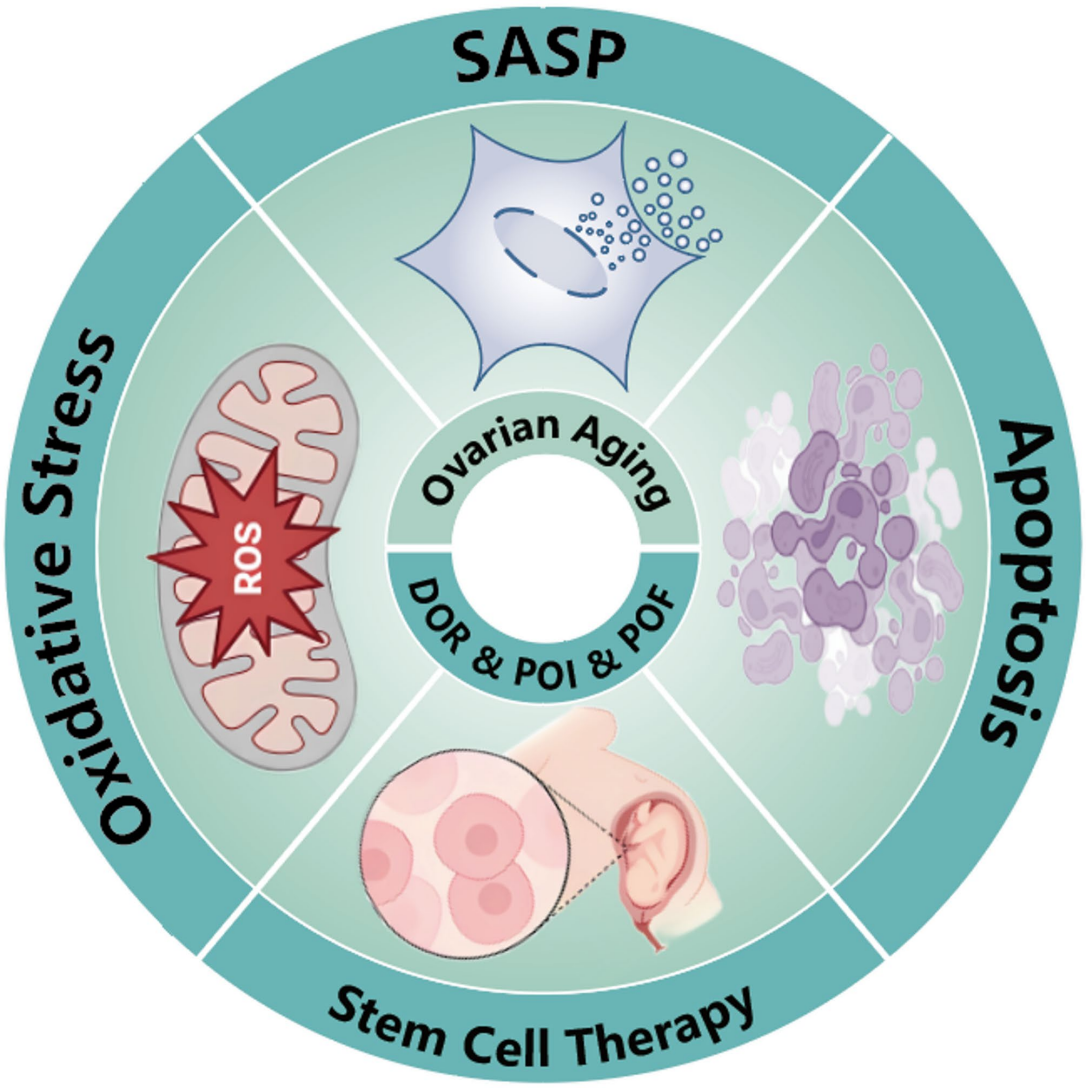
The roles of MAPK pathway in Ovarian aging and DOR/POI/POF

Current studies into ovarian aging highlights the significant role of the MAPK signaling pathways in mediating oxidative stress, apoptosis, and the senescence-associated secretory phenotype (Fig. 3). Targeting oxidative stress through antioxidant treatments, such as melatonin and NAC, may mitigate the activation of MAPK pathways associated with cellular dysfunction and apoptosis in GCs. Furthermore, the therapeutic potential of mesenchymal stem cell transplantation in enhancing MAPK signaling suggests a promising avenue for restoring ovarian function.

### MAPK in DOR/POI/POF

Diminished Ovarian Reserve (DOR) refers to a reduced capacity of the ovaries to produce oocytes or hormones. Premature Ovarian Insufficiency (POI), previously known as premature ovarian failure, is characterized by the loss of normal ovarian function before age 40, leading to irregular menstrual cycles or amenorrhea and often associated with autoimmune disorders or genetic anomalies. Premature Ovarian Failure (POF) is an older term similar to POI, but it emphasizes the cessation of ovarian function rather than its gradual decline. In short, all three exhibit premature ovarian dysfunction.

Increasing evidence reveals that the activation of the MAPK signaling pathway is involved in the occurrence of DOR/POI/POF, also regarded as the anomalous ovarian aging (Fig. 3). The role of the MAPK pathway and its associated factors in DOR/POI/POF is multifaceted, including the regulation of inflammatory responses, oxidative stress, cell proliferation, apoptosis, and sex hormone levels [233, 267, 268]. The MAPK signaling pathway is one of several key pathways associated with Fragile X-associated Primary Ovarian Insufficiency (FXPOI) in carriers of FMR1 premutation, and it has been found to be downregulated in women with FXPOI [269]. In rats with cyclophosphamide-induced POF, a significant increase in the phosphorylation levels of p38 MAPK and NF-κB p65 has been observed [267], alongside the activation of the MEK1/2 and ERK1/2 signaling pathways [270]. Additionally, Tripterygium wilfordii has been shown to significantly upregulate the expression of apoptotic proteins, including cleaved caspase-3 and Bax, in the ovaries of POF rats through the activation of MAPK signaling pathways, including ERK1/2, JNK, and p38 [271]. Furthermore, Bisphenol can increase oxidative stress levels in mouse oocytes while decreasing p-MAPK protein levels, thereby disrupting meiotic spindle assembly, inhibiting oocyte maturation, and ultimately impairing ovarian function [272].

MAPK1 plays a crucial role in regulating the expression of LH β-subunit and FSH β-subunit, as well as in oocyte meiotic maturation and ovulation [45, 268]. The FSHR variant p.Ala462Pro nearly completely abolishes ERK activation, while the p.Ala621Val variant retains 45.79% and 33.21% of maximal ERK activation levels after 5 and 10 min of FSHR agonist treatment, respectively. This indicates that the ERK signaling pathway is a significant marker for assessing FSHR functionality and may play a role in the pathogenesis of POI [273]. Mutations in the FSH receptor, such as p.L597I, may affect ovarian function by influencing the activation of the MAPK pathway, particularly the phosphorylation of ERK1/2, potentially contributing to the development of POI [274]. Additionally, mutations I423T and D408Y in FSHR have been shown to impact FSH-induced ERK1/2 phosphorylation, which could adversely affect ovarian function, leading to POF [275]. Furthermore, *miR-16-5p* is upregulated in GCs from the normal ovarian reserve (NOR) group, targeting MAP2K1 and MAPK3 kinases, which are integral components of the ERK pathway associated with the expression of acute regulatory proteins in steroidogenesis. Therefore, the downregulation of *miR-16-5p* may decrease steroid production in GC from women with DOR by enhancing the activity of MAP2K1 and MAPK3 [276].

In rat models of POI induced by high-fat high-sugar (HFHS) diets and radiation [233, 277, 278], both p38 and JNK have been shown to play critical roles. In established mouse models of POI, treatment with pZP3 resulted in elevated levels of p38 and p-JNK [279]. Advanced oxidation protein products (AOPPs) activate the ROS-JNK/p38 MAPK-p21 signaling pathway, leading to cell cycle arrest of ovarian GCs, which may be an important aspect of POI pathogenesis [229]. Furthermore, following AOPP treatment of KGN cells, there was a significant increase in the phosphorylation levels of JNK and p38 MAPK. Pre-treatment of KGN cells with the JNK inhibitor SP600125 and the p38 MAPK inhibitor SB203580 alleviated AOPP-induced G1/G0 phase cell cycle arrest, while enhancing the expression of cell cycle-related proteins cyclin E1 and CDK2, and reducing p21 expression [229]. In GCs from patients with biochemical early primary ovarian insufficiency (bPOI), reduced expression levels of RAC1 were observed. In RAC1 knockdown cells, P21, a molecule associated with cell cycle and apoptosis, was upregulated, which is consistent with the aforementioned findings [280]. *Wdr62* promotes the initiation of meiotic division in mouse oocytes by activating the JNK signaling pathway, and mutations in *Wdr62* may be associated with the occurrence of POI [281]. Overexpression of *miR-146b-5p* alleviated the POI phenotype induced by high glucose and high fat in mice by inhibiting the Dab2ip/Ask1/p38 MAPK signaling pathway and the phosphorylation of γH2A.X [282]. In the pathogenesis of DOR, downregulation of *miR-106a* led to increased expression of ASK1, which in turn activated the p38 MAPK signaling pathway and promoted GCs apoptosis [283, 284].

The role of the JNK pathway in POI remains controversial. In mice treated with the JNK inhibitor SP600125, a decrease in GC activity and an increase in apoptosis were observed, whereas treatment with anisomycin (a JNK agonist) promoted GC activity and reduced apoptosis [285]. This discrepancy may be related to the involvement of the JNK signaling pathway in the regulation of autophagy. The expression of HO-1 has been shown to promote autophagy through the activation of the JNK/Bcl-2 signaling pathway, which helps restore ovarian function in POF mice. SP600125 may inhibit the JNK pathway, thereby affecting the autophagic process and resulting in decreased GC activity and increased apoptosis [285]. Consequently, future clinical practices should consider the interplay between apoptosis and autophagy, as well as the potential crosstalk between the MAPK pathway and other signaling pathways.

In recent years, significant progress has been made in the intervention studies targeting MAPK pathway-related factors for the treatment of POI, providing new insights for clinical management of affected patients. Through protein-protein interaction (PPI) network analysis, MAPK1 has been identified as one of the common core targets for Zuo Gui Wan and You Gui Wan in the treatment of POI [286]. Jin Feng Wan demonstrated therapeutic effects on cyclophosphamide-induced POI in rats by inhibiting the IL-17 A/IL-6 axis and reducing the activation of MEK1/2 and ERK1/2 signaling pathways [270]. The MAPK signaling pathway plays a critical role in the pathogenesis of POI induced by ovariectomy in rats, and Qi Lin Wan may help mitigate follicular depletion by suppressing the overactivation of MAPK and PI3K-AKT pathways, thereby exerting a therapeutic effect on POI [287]. Similarly, Sijunzi Decoction may exert its therapeutic effects on POI by influencing the PI3K/Akt and MAPK/ERK signaling pathways [288]. Bushen Huoxue decoction improved follicular development in aging mice with reduced ovarian reserve by increasing the protein levels of p-ERK1/2 and activating ERK1/2 phosphorylation, which also enhanced the proliferation of GCs and oocyte complexes [289]. Melatonin promoted follicle growth and proliferation following ovarian damage induced by cadmium in DOR rats through the ERK1/2 and mTOR pathways [290, 291]. Furthermore, treatment with pearl powder significantly reduced the phosphorylation levels of ERK1/2, JNK, and p38 in the ovaries of POI rats, which correlated with decreased expression of apoptotic proteins caspase-3 and Bax [271].

In mice, myrcene has been shown to counteract the elevated levels of p-p38 and p-JNK induced by pZP3 treatment [279]. Chrysin treatment effectively reduced the phosphorylation levels of p38 and JNK in radiation-induced rats [233]. Additionally, NAC treatment was also able to inhibit the activation of p38 and JNK induced by radiation, leading to a reduction in apoptosis of ovarian GCs and thereby providing protective effects on ovarian function [277]. Both Erxian Decoction and Jian-Pi-Yi-Shen decoction decoction demonstrated potential protective effects in POF rats by suppressing the activation of JNK and improving mitochondrial function [292, 293].

In a DOR mouse model, cyclophosphamide treatment resulted in downregulation of p38 phosphorylation, while Bu Shen Tiao Chong recipe and DHEA treatments were able to reverse this effect [294]. Heat-transformed saponin (HTS) provided protective effects on POF model rats and KGN cells by inhibiting the activation of the p38 MAPK/NF-κB p65 signaling pathway, thereby reducing inflammation and oxidative stress [267]. In a DOR rat model induced by triptolide, miRNA-KEGG network analysis revealed that differentially expressed miRNAs after acupuncture treatment had significant associations with the MAPK signaling pathway [295]. The effects of acupuncture on POF were comparable to those of estrogen, possibly linked to the inhibition of p38 MAPK protein expression, which affects the activation of the p38 MAPK signaling pathway and reduces apoptosis of GCs in rats [296].

Excitingly, promising stem cell therapies may also exert their effects partially through the MAPK pathway. Transplantation of umbilical cord mesenchymal stem cells (UCMSCs) has been shown to significantly increase the expression of p-ERK1/2 in a POF pig model while markedly reducing the apoptosis rate of ovarian cells in these models [297]. Human amniotic epithelial stem cells (hAESCs) promote the proliferation of mouse GCs and inhibit cell apoptosis by activating the AKT and ERK signaling pathways, providing a reference for the treatment of POI [298]. Moreover, UCMSCs transfected with HO-1 enhance the activity of mouse GCs and reduce their apoptosis, contributing to the restoration of ovarian function in POF mice, which is associated with the activation of the JNK/Bcl-2 signaling pathway. JNK can activate autophagy by interfering with the Bcl-2/Beclin-1 complex, coinciding with an increase in serum IL-10 levels and an elevation of CD8+CD28−T cells in POF mice [285]. Additionally, UCMSC treatment leads to increased ovarian size, a rise in the number of primary and secondary follicles, and a decrease in the number of atretic follicles in POF mice. Notably, the phosphorylation level of p38 MAPK is enhanced following UCMSC treatment [299].

### MAPK in PCOS

**Fig. 4:**
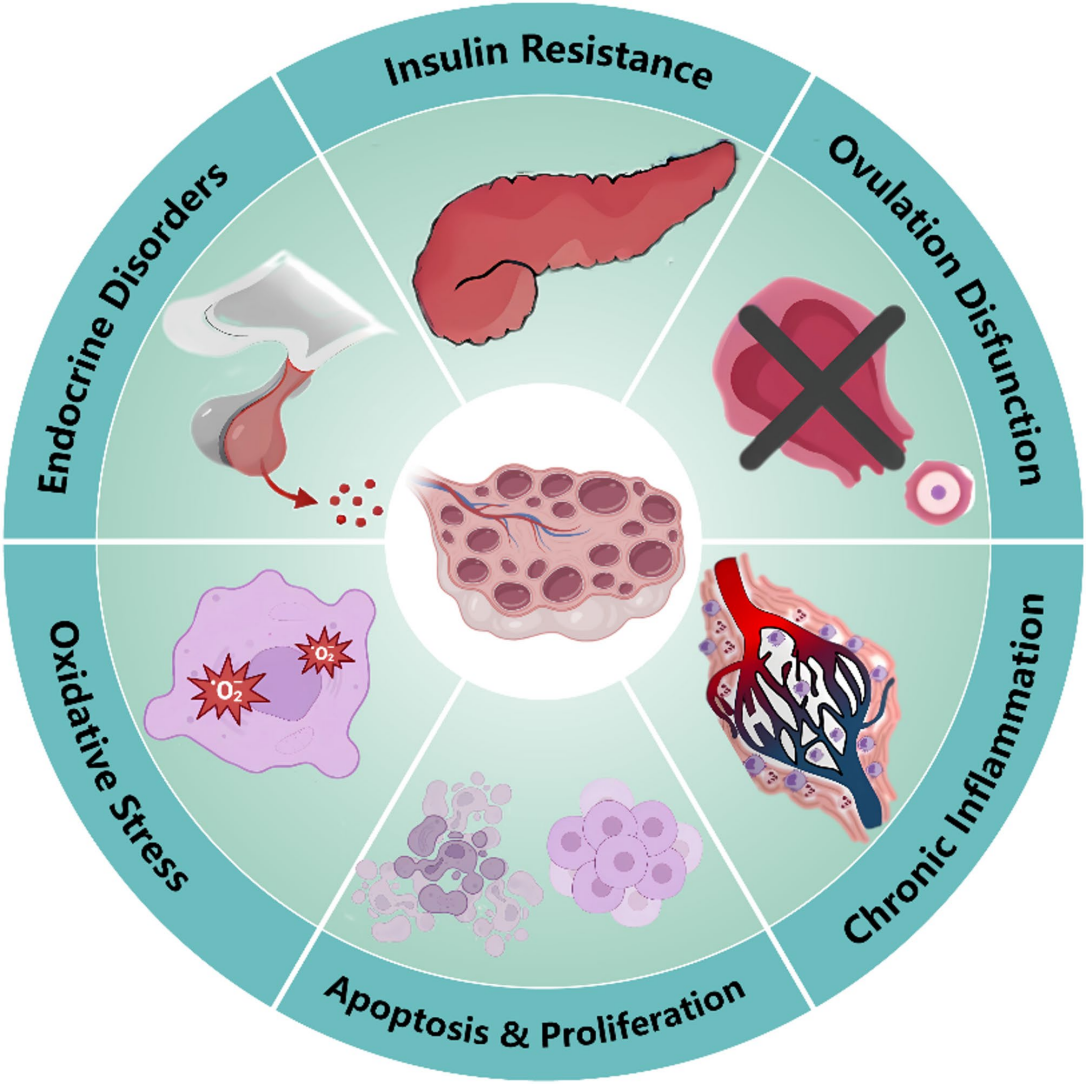
The roles of MAPK pathway in PCOS

Polycystic Ovary Syndrome (PCOS) is a common endocrine disorder characterized by hormonal imbalances, irregular menstrual cycles, and polycystic ovaries, often associated with insulin resistance and metabolic disturbances. The MAPK signaling pathway plays a crucial role in mediating various pathogenic mechanisms of PCOS. Endocrine disruption can arise from altered signaling in ovarian follicles, affecting hormone production. Insulin resistance, prevalent in PCOS, activates the MAPK pathway, enhancing ovarian androgen synthesis. Impaired ovulation results from disrupted follicular development, linked to MAPK-driven gene expression changes. Oxidative stress is exacerbated by dysregulated MAPK signaling, leading to increased ROS. Additionally, an imbalance between cell proliferation and apoptosis in ovarian tissues can be influenced by MAPK activity, promoting cyst formation. Lastly, chronic inflammation associated with PCOS is modulated by MAPK, which mediates the inflammatory response, further contributing to the disease’s pathogenesis (Fig. 4).

#### Endocrine disorders

Abnormal MAPK signaling pathways contribute to the excessive androgen production in the ovaries of patients with PCOS [300–302]. In the ovarian stromal cells of PCOS patients, reduced activity of MEK1/2 and ERK1/2 is associated with increased androgen production, independent of insulin levels [303]. Introducing dominant negative MEK1 (DN-MEK1) into normal human follicular stromal cells resulted in increased abundance of *CYP17* mRNA and dehydroepiandrosterone [303]. In PCOS patients, decreased levels of *miR-125b-5p* lead to increased expression of *PAK3* [304], subsequently promoting ERK1/2 activation, which further influences the expression of steroidogenic genes in letrozole-induced mouse GCs and TCs. In PCOS, ERK1/2 activation increases the expression of androgen synthesis-related genes, such as *Cyp17a1* and *Cyp11a1*, leading to elevated testosterone secretion. Concurrently, it decreases the expression of estrogen synthesis-related genes, such as *Cyp19a1* and *Hsd17b1*, resulting in reduced estradiol secretion [304]. GCs treated with metformin exhibit significantly suppressed mRNA expression and activity of aromatase P450, both at basal levels and under insulin stimulation, an effect mediated through the activation of the MEK/ERK pathway [305]. Elevated serum levels of testosterone, LH, LH/FSH ratio, and HOMA-IR index were observed in PCOS rat models, alongside significantly increased expression levels of MIF, p-JNK, and p-p38 in ovarian tissues compared to control groups [306]. In bovine ovarian stromal cells, the activation of p38 and JNK is involved in regulating the expression of CYP17A1 and PAI-1, both of which are associated with hyperandrogenemia and ovarian fibrosis in PCOS [99].

Interestingly, recent studies have shown that the levels of HB-EGF protein in the follicular fluid of PCOS women are elevated compared to non-PCOS women. HB-EGF promotes the production of more estrogen in GCs by binding to its receptor EGFR, activating the cAMP-PKA-JNK/ERK signaling pathway. The use of JNK inhibitor SP600125 and ERK inhibitor GDC-0994 can block the stimulatory effects of HB-EGF on estrogen synthesis and CYP19A1 expression. Thus, the increase in estrogen may represent a compensatory response of the body to the endocrine disorders in PCOS patients. The roles of various factors in the MAPK pathway and the JNK/ERK-mediated increase in estrogen in PCOS warrant further investigation.

#### Insulin resistance

In women with PCOS, gene polymorphisms of ERK1/2 are associated with insulin resistance [307–310]. Recent studies have identified that *miR-612* regulates the MAPK pathway by targeting *Rap1b*, which may have significant implications for insulin resistance [302]. In bovine GCs, the MAPK pathway appears to be linked to the expression of IGF1R, which plays a critical role in the pathogenesis of PCOS [311]. IGF-1-induced DNA synthesis in GCs is enhanced via the MAPK pathway, while insulin pretreatment can attenuate IGF-1-induced cell proliferation and Akt phosphorylation, although it does not affect ERK1/2 phosphorylation [312].

Activation of the MAPK pathway may also lead to a decrease in GLUT4 (glucose transporter type 4) expression, thereby impairing glucose transport [306, 313]. Furthermore, activation of the MAPK pathway can inhibit the activation of insulin receptor substrates (IRS), contributing to the development of insulin resistance. Within the context of PCOS, this insulin resistance is linked to abnormal glucose metabolism [314]. Berberine treatment has been shown to elevate GLUT4 mRNA and protein levels in PCOS rats, which may relate to its inhibitory effects on the MAPK pathway. Additionally, berberine treatment significantly reduced the expression of MAPK signaling proteins in PCOS rats, suggesting that it may alleviate insulin resistance by inhibiting MAPK pathway activation [315]. Both metformin and pioglitazone treatments have been found to suppress JNK phosphorylation in PCOS rats, indicating that these drugs may improve insulin resistance through the inhibition of JNK activity [316].

#### Ovulatory disorders

Genetic inactivation of ERK1/2 in mouse GCs leads to ovulatory disorders and infertility [120, 310]. Knockdown of *EPHA7* in KGN cells results in decreased phosphorylation levels of ERK1/2, subsequently reducing the expression of *C/EBPβ*,* KLF4*, and *ADAMTS1* [177].

In patients with PCOS, there is a significant reduction in the proportion of CD24(+) GCs, accompanied by decreased mRNA levels of *CD24*,* PTGS2*,* SLCO2A1*,* PTGES*,* ARK1C1*,* PLA2G4A*, and *ABCC4* in GCs [168]. Knockdown of CD24 leads to decreased stability of EGFR protein and reduced phosphorylation levels of EGFR, as well as its downstream effector ERK1/2 [168]. In PCOS patients, the expression level of FHL2 is upregulated, which interacts with ERK1/2 to inhibit its phosphorylation. Since activation of ERK1/2 is essential for ovulation, this inhibitory effect of FHL2 may block the normal ovulatory process, leading to ovulatory disorders [317].

Glutamine, through glutaminyl-tRNA synthetase (QARS) catalyzed glutamylation at the ASK1 K688 site, inhibits ASK1 activation, thereby reducing phosphorylation levels of JNK and p38, which may help decrease apoptosis in mouse GCs [318]. In PCOS patients, the reduced rate of GC apoptosis may result in incomplete rupture of the follicular wall, obstructing the normal ovulation process [318]. Overexpression of PGC-1α significantly inhibits ox-LDL and LH-induced p38 activation, thereby protecting human GCs from damage and improving ovulatory disorders [319]. In mouse COCs treated with testosterone, there is an observed upregulation of ovulation-related gene expression, which can be reversed by treatment with metformin, si-p38 MAPK, or the ER stress inhibitor TUDCA [320].

#### Oxidative stress

In GCs of patients with PCOS, a significant increase in ROS production has been observed, correlating with the activation of p38 MAPK [319]. In a dihydrotestosterone (DHT) treated PCOS mouse model, phosphorylation levels of p38 MAPK in ovarian GCs and COCs were found to be elevated [320]. Overexpression of PGC-1α was shown to significantly inhibit ROS production and p38 activation induced by ox-LDL and LH. Treatment with oxidants significantly suppressed ox-LDL and LH-induced p38 activation, while p38 inhibitors did not significantly affect ROS production induced by these factors, indicating that ROS acts as an upstream molecule in the p38 signaling pathway during ox-LDL and LH-induced damage in human GCs [319]. In primary cultured GCs and COCs, testosterone-induced ER stress can be alleviated by treatment with metformin or the p38 MAPK inhibitor SB203580 [320]. In bovine ovarian membrane cells, inhibition of autophagy may lead to mitochondrial dysfunction and ROS generation, subsequently activating p38 and JNK pathways, which may contribute to the development of PCOS [321].

In PCOS, the expression level of the P2X7 receptor is increased, which can be activated by binding to ligands such as ATP. Activation of the P2X7 receptor promotes the aggregation and activation of NADPH oxidase 2 (NOX2), a key enzyme responsible for intracellular ROS production and critical for maintaining cellular redox balance. The P2X7 antagonist A740003 reduces the expression of NOX2 and ROS production, leading to diminished activation of the JNK signaling pathway in mouse GCs, resulting in decreased production of inflammatory factors and reduced cell apoptosis [322]. Upregulation of Map4k4 expression in rats activates the JNK/c-JUN signaling pathway, leading to oxidative stress and damage in GCs. *miR-185-5p* may alleviate the pathological state of PCOS by lowering Map4k4 expression [323].

Overexpression of Kisspeptin in KGN cells increases phosphorylation levels of ERK while reducing the accumulation of ROS. Treatment with PD98059 reverses the reduction of ROS levels induced by Kisspeptin, indicating that the ERK signaling pathway plays a role in Kisspeptin’s regulation of oxidative stress [324]. Vitamin D3 may improve mitochondrial dysfunction and oxidative stress in PCOS mouse models through the ERK1/2 signaling pathway, thereby positively influencing the function of GCs [325].

#### Imbalance between proliferation and apoptosis

The ERK pathway plays a critical role in regulating the survival, proliferation, and differentiation of GCs. Through the construction of luciferase reporter vectors containing wild-type and mutant 3’ UTRs of *Rap1b* mRNA, experiments have shown that miR-612 can directly bind to the 3’ UTR of *Rap1b* mRNA, inhibiting its expression. *Rap1b*, a small GTP-binding protein, influences various cellular behaviors, including migration, invasion, and proliferation, by inhibiting MAPK activation [302]. In rats, the ERK1/2 pathway plays an important role in ovarian GCs proliferation, with its activation potentially linked to the morphological changes observed in the ovaries of PCOS models [326].

Treatment of KGN cells with the ERK1/2 antagonist PD98059 inhibits Kisspeptin-enhanced cell proliferation and eliminates the anti-apoptotic effects of Kisspeptin, indicating that Kisspeptin’s pro-proliferative and antioxidant effects on KGN cells are at least partially mediated through the ERK signaling pathway [324]. Overexpression of Kisspeptin results in reduced apoptosis in KGN cells, with upregulation of the anti-apoptotic factor Bcl-2 and downregulation of the pro-apoptotic factors caspase-3 and Bax [324]. Additionally, overexpression of FDPS enhances the expression of PCNA and Ki67 in ovarian tissue and promotes GCs proliferation through the phosphorylation of p38 and ERK, thereby improving ovarian dysfunction in PCOS mice [327]. In KGN cells, HB-EGF induces mPTP opening via the cAMP-PKA-JNK/ERK-Ca^2+^-FOXO1 pathway, resulting in the release of cytochrome C from mitochondria to the cytosol and triggering apoptosis in GCs[99]. Vitamin D3 and MAPK activators enhance the phosphorylation of MAPK-ERK1/2 in GCs, reducing the expression of the pro-apoptotic gene BAX while upregulating the anti-apoptotic gene BCL-2 [325].

In vitro studies have shown that silencing Map4k4 (which inhibits JNK protein activation) improves the viability of GCs derived from PCOS rats, reducing apoptosis rates and influencing the expression of Bax and Bcl-2 proteins [323]. Map4k4 may be a target gene of miR-185-5p, which is expressed at significantly lower levels in PCOS GCs compared to normal GCs; overexpression of miR-185-5p reduces Map4k4 expression [323]. In PCOS patients, especially those who are obese, the expression of PGC-1α is significantly decreased, accompanied by a notable increase in GCs apoptosis [319]. Pre-treatment with antioxidants (such as NAC and Tempol) and p38 inhibitors (such as SB203580 and SB202190) significantly reduces apoptosis induced by ox-LDL and LH in human GCs [319].

However, some studies have measured the effects of hyperandrogenism on apoptosis, finding that in GCs of DHT-treated mice, the rate of apoptosis and apoptotic signaling only slightly increased, with no significant difference compared to the control group [320].

#### Chronic inflammation

The MAPK signaling pathway and its associated factors may mediate the pathogenesis of PCOS through inflammatory responses [306]. In KGN cells, activation of the MAPK pathway is linked to the activation of inflammasomes and the enhancement of inflammatory responses [328]. A study conducted on the Turkish population found that the levels of the inflammatory marker CRP were significantly higher in PCOS patients compared to the control group, with CRP levels positively correlating with BMI, HOMA-IR, and total testosterone levels [310]. In mice, activation of the P2X7 receptor leads to increased expression of pro-inflammatory cytokines, such as tumor necrosis factor α (TNF-α), interleukin 1β (IL-1β), and interleukin 6 (IL-6), via the NOX2/JNK signaling pathway [322]. The JNK pathway plays a critical role in the inflammation and fibrosis processes associated with PCOS, and the application of the JNK inhibitor SP600125 may offer a new therapeutic strategy for the prevention of PCOS [321, 329].

Moreover, the MAPK pathway and its factors significantly influence angiogenesis in PCOS. Metformin reduces angiogenesis by modulating these pathways and their downstream factor TSP-1. In women with PCOS, serum levels enhance endothelial cell migration and angiogenesis; these effects are significantly diminished following metformin treatment, and are further countered when TSP-1 neutralizing antibodies are administered, indicating that TSP-1 may regulate angiogenesis by influencing the MAPK pathway [330].

In summary, PCOS is a multifactorial and highly heterogeneous disorder, and the modeling approaches and sample selection may contribute to the variability in research outcomes. Consequently, many questions remain to be clarified in current studies. For example, how can we establish a definitive relationship between the activation of the MAPK pathway and the various pathogenic mechanisms of PCOS? What role does MAPK-mediated estrogen elevation play in the pathophysiology of PCOS? Furthermore, how can we achieve a balance between proliferation and apoptosis by influencing the MAPK pathway to improve the phenotypic manifestations of PCOS?

### MAPK in OHSS

Ovarian Hyperstimulation Syndrome (OHSS) is a potentially serious condition that can occur in women undergoing fertility treatments, particularly those involving ovulation induction and in vitro fertilization (IVF). It is characterized by an exaggerated response to hormonal stimulation, leading to the enlargement of the ovaries, increased vascular permeability, and fluid accumulation in the abdominal cavity, which can cause abdominal distension and discomfort.

In the follicular fluid of individuals with and without OHSS, several microRNAs (miRNAs) exhibit differential expression, including those associated with the MAPK signaling pathway [331, 332]. Analysis of potential target genes for these differentially expressed miRNAs, predicted using Target Scan 6.2 and miRDB algorithms, indicates that the MAPK signaling pathway is significantly enriched, which suggests that miRNAs may regulate the MAPK pathway in OHSS, thereby influencing ovarian responsiveness [332]. The role of the ERK pathway in OHSS primarily relates to its function in regulating GCs proliferation and apoptosis. In patients with OHSS, the p-ERK is upregulated, likely associated with elevated levels of inflammatory factors such as vascular endothelial growth factor (VEGF) [331]. Under normal physiological conditions, the activation of ERK1/2 promotes the expression of SPRY2, which, in turn, limits EGFR-mediated signaling by inhibiting the ERK1/2 pathway, thus establishing a negative feedback mechanism. However, in the context of OHSS, this balance may be disrupted, leading to excessive expression of SPRY2 and over-inhibition of the COX-2/PGE2 pathway [333]. miR-27 may exert its effects on the pathological processes of OHSS by targeting SPRY2 to inhibit the ERK signaling pathway [331].

The ERK1/2 and p38 MAPK signaling pathways are critical mediators of TGF-β1-induced expression and secretion of VEGF. Inhibiting these signaling pathways may offer a potential therapeutic strategy to alleviate the symptoms of OHSS [334]. Lysophosphatidic acid activates the MAPK pathway through its receptors, which in turn enhances the expression of IL-8 and IL-6 in granulosa-lutein cells. These cytokines are associated with angiogenesis and inflammatory responses, suggesting their pivotal role in the pathology of OHSS [335]. When ovarian endothelial cells were treated with Hyper IL-6, a complex of IL-6 and soluble IL-6 receptor alpha, an increase in VEGF expression and enhanced vascular permeability were observed. Pre-treatment with PD98059 effectively prevented the increase in vascular permeability induced by Hyper IL-6, providing direct evidence of the MAPK pathway’s involvement in IL-6-mediated vascular leakage [336]. Moreover, in rat models, electroacupuncture has been reported to mitigate the progression of OHSS by modulating angiogenesis-related signaling pathways, including the MAPK pathway [337]. In COH mice, luteal angiogenesis was found to be affected, while Bushen Huoxue recipe treatment was able to promote luteal vascularization, potentially through the LHCGR-MEK1/2-ERK1/2-VEGFA/FGF2 signaling cascade [338].

Current studies indicates while differential expression of miRNAs and the involvement of the ERK pathway are acknowledged, the precise molecular mechanisms linking these factors to OHSS remain inadequately explored. Additionally, the therapeutic potential of targeting the MAPK pathway is promising, but the efficacy and safety of such interventions require further investigation in clinical settings. There is also a need for comprehensive studies that assess the interplay between MAPK and other signaling pathways in the context of OHSS.

## Concluding remarks and looking forward

In this review, we have explored the intricate roles of the MAPK signaling pathway throughout various stages of ovarian folliculogenesis. From primordial follicle formation to ovulation and luteinization, MAPK signaling emerges as a fundamental player, orchestrating a multitude of cellular processes critical for ovarian function. The physiological relevance of this pathway is underscored by its involvement in primordial follicle activation, steroidogenesis, and the selection of dominant follicles, among other processes. These functions highlight the MAPK pathway as not merely a signaling cascade but as a central hub that integrates hormonal signals, environmental cues, and intrinsic cellular responses to ensure the proper development and maturation of ovarian follicles. However, as we delve deeper into our understanding of MAPK signaling, it becomes evident that there remain significant gaps in our knowledge, particularly concerning its precise mechanisms and interactions with other signaling pathways. For instance, while we have identified the roles of MAPK in primordial follicle activation and steroidogenesis, the exact molecular interactions that drive these processes require further elucidation. Similarly, the cross-talk between MAPK and other signaling pathways, such as PI3K/Akt and TGF-β, remains an area ripe for exploration. Understanding these interactions could provide critical insights into the fine-tuning of follicular development and the prevention of ovarian dysfunction.

The pathophysiological implications of dysregulated MAPK signaling are equally critical, particularly in conditions such as POI, PCOS, and OHSS. In POI, for instance, the aberrant activation of the MAPK pathway may contribute to the loss of ovarian function, but the exact nature of this dysregulation remains poorly characterized. Future research should aim to establish a clearer relationship between MAPK signaling and the etiology of POI to develop targeted therapeutic strategies. Similarly, in PCOS, the hyperactivation of MAPK signaling is thought to play a role in various pathogenic mechanisms. Investigating how MAPK signaling can be modulated in PCOS patients could lead to novel treatment options that address both the hormonal imbalances and the ovarian dysfunction characteristic of this condition.

Looking forward, a multi-faceted approach to research in this area will be essential. Integrating advances in genomic, proteomic, and metabolomic technologies could yield a more comprehensive understanding of the MAPK signaling pathway in ovarian physiology and pathology. Moreover, the application of systems biology approaches to decipher the complex networks in which MAPK signaling operates will be vital [339]. Understanding the broader context in which MAPK functions—considering factors like cellular microenvironments, hormonal influences, and genetic predispositions—could lead to breakthroughs in our understanding of ovarian biology. The development of advanced animal models and in vitro systems will also enhance our ability to study MAPK signaling dynamics in a controlled environment. These models can provide valuable insights into the temporal and spatial regulation of MAPK activity within the ovarian follicle, thereby elucidating how disruptions in this signaling cascade can lead to dysfunction. Furthermore, innovations in imaging techniques may allow for real-time visualization of MAPK signaling events in living tissues [340], providing an unprecedented opportunity to observe how this pathway drives folliculogenesis in real-time.

In summary, the MAPK signaling pathway plays a pivotal role in ovarian folliculogenesis, influencing both physiological and pathological processes. However, many aspects of this signaling cascade remain to be fully understood. Continued research in this area is essential not only for advancing our fundamental knowledge of ovarian biology but also for developing innovative therapeutic strategies to address the myriad of ovarian disorders that affect women’s health. As we look forward, embracing a holistic and interdisciplinary approach will be key to unlocking the full potential of MAPK signaling research in the field of reproductive medicine.

Despite the rapid accumulation of reports on the contribution of the MAPK pathway to ovarian folliculogenesis, a number of key questions remain unanswered and new questions continue to emerge as new information becomes available. Some of the key questions for further research into this emerging signaling pathway are listed here:

1. Where are MAPK factors located in ovarian folliculogenesis? The localization and roles of MAPKK and MAPKK, the core kinases of the MAPK pathway, at various stages of follicular development, ovulation, luteinization, and degeneration are unknown. Studies have shown that under most conditions, dephosphorylated ERK, JNK and P38 are enriched in the cytoplasm and phosphorylated ERK, JNK and P38 are present in the nucleus. However, the locations where ERK, JNK and P38 phosphorylation and dephosphorylation occur remain to be determined. In the future, we can employ single-cell techniques as well as Spatial Omics to further characterize the cellular and subcellular localization of MAPK factors [341].
2. Even though atypical MAPK factors have received widespread attention, their role in ovarian folliculogenesis remains little reported. What are the specific roles of atypical MAPK factors in different stages of folliculogenesis? How do these factors integrate with canonical MAPK signaling? How do non-traditional MAPK factors respond to changes in the ovarian microenvironment, such as during stress or inflammation, and how do they influence follicular health? What roles do atypical MAPK factors play in ovarian disorders such as PCOS, POI, or OHSS? Could they serve as potential therapeutic targets?
3. MAPK pathway factors function through transcription-dependent and non-transcription-dependent pathways. However, few studies have delved into MAPKAPKs and transcription factors downstream of the MAPK pathway. Ovarian folliculogenesis-associated MAPKAPKs and transcription factors urgently need to be characterized by more in-depth experiments.
4. Almost all signaling pathways play a role in ovarian folliculogenesis, but the interplay among individual pathways, especially between the MAPK pathway and various other pathways, has not been drilled down. The interaction between MAPK signaling and other pathways (such as PI3K/Akt, TGF-β, and Notch) during follicle development remains unclear. Understanding these interactions is vital for a comprehensive view of ovarian physiology.
5. The regulatory role of non-coding RNAs(ncRNA) (e.g., miRNAs, lncRNAs) in modulating MAPK signaling during folliculogenesis is not fully characterized. How do specific non-coding RNAs interact with MAPK pathway components during folliculogenesis? What are the upstream regulators of ncRNAs involved in the MAPK signaling during folliculogenesis? What are the mechanisms through which ncRNAs regulate the expression of key MAPK-related genes?
6. Previously, studies of cell death associated with ovarian folliculogenesis have focused on apoptosis and autophagy. As various novel modes of cell death continue to be proposed and defined, the roles of ferroptosis, pyroptosis, and cuproptosis in ovarian folliculogenesis have begun to be extensively studied. What are the novel cell death pathways that have yet to be characterized in the context of ovarian folliculogenesis, and how are they regulated by MAPK signaling? Can interventions targeting specific MAPK pathways modulate these cell death mechanisms to improve follicular health and fertility outcomes?

## Data Availability

No datasets were generated or analysed during the current study.
